# Optimizing the delivery of self-disseminating vaccines in fluctuating wildlife populations

**DOI:** 10.1371/journal.pntd.0011018

**Published:** 2023-08-18

**Authors:** Courtney L. Schreiner, Andrew J. Basinski, Christopher H. Remien, Scott L. Nuismer

**Affiliations:** 1 Department of Ecology and Evolutionary Biology, University of Tennessee, Knoxville, Tennessee, United States of America; 2 Institute for Interdisciplinary Data Sciences, University of Idaho, Moscow, Idaho, United States of America; 3 Department of Mathematics and Statistical Sciences, University of Idaho, Moscow, Idaho, United States of America; 4 Department of Biological Sciences, University of Idaho, Moscow, Idaho, United States of America; George Washington University School of Medicine and Health Sciences, UNITED STATES

## Abstract

Zoonotic pathogens spread by wildlife continue to spill into human populations and threaten human lives. A potential way to reduce this threat is by vaccinating wildlife species that harbor pathogens that are infectious to humans. Unfortunately, even in cases where vaccines can be distributed en masse as edible baits, achieving levels of vaccine coverage sufficient for pathogen elimination is rare. Developing vaccines that self-disseminate may help solve this problem by magnifying the impact of limited direct vaccination. Although models exist that quantify how well these self-disseminating vaccines will work when introduced into temporally stable wildlife populations, how well they will perform when introduced into populations with pronounced seasonal population dynamics remains unknown. Here we develop and analyze mathematical models of fluctuating wildlife populations that allow us to study how reservoir ecology, vaccine design, and vaccine delivery interact to influence vaccine coverage and opportunities for pathogen elimination. Our results demonstrate that the timing of vaccine delivery can make or break the success of vaccination programs. As a general rule, the effectiveness of self-disseminating vaccines is optimized by introducing after the peak of seasonal reproduction when the number of susceptible animals is near its maximum.

## Author summary

Pathogens such as Ebola, rabies, and Lassa virus that usually infect wildlife can jump to the human population. In the worst case, this can lead to outbreaks or pandemics such as happened in 2014 with Ebola and 2019 with SARS-CoV-2. One approach to mitigate the threat of pathogens spilling into the human population is to proactively vaccinate wildlife harboring these pathogens before the pathogens infect humans. With traditional vaccines, administering enough vaccines to the wildlife population to limit pathogen spread is challenging. To address this challenge, recent technological advances have allowed the development of vaccines that allow some degree of spread of the vaccine from animal to animal. However, for a vaccination campaign using these self-disseminating vaccines to be implemented successfully, we need to know when vaccines should be administered. We used mathematical models to explore how the reservoir host’s population ecology and properties of the vaccine affect the success of a vaccination campaign. Our results demonstrate that the timing of vaccine delivery relative to seasonal reproduction can make or break the success of vaccination programs. The effectiveness of self-disseminating vaccines is optimized by introducing vaccine after the peak of seasonal reproduction when the number of animals available for vaccination is highest.

## Introduction

The majority of human infectious diseases are caused by pathogens with animal origins [1]. As the human population continues to encroach on wildlife habitat, zoonotic pathogens such as Ebola virus, *Borrelia burgdorferi*, Lassa virus, Sin Nombre virus, and Nipah virus pose an increasing threat of spillover into the human population [1–5]. Several of these emerging infectious diseases have had devastating impacts on public health. The 2014 Ebola outbreak, for example, killed more than 11,000 people [5], and the ongoing SARS-CoV-2 pandemic has killed millions [6]. The SARS-CoV-2 pandemic has made the perils of our current reactionary approach to managing emerging infectious disease clear and helped to focus attention on methods that proactively reduce the risk of spillover and emergence.

Vaccinating wildlife reservoir populations is a proven method for lowering pathogen prevalence and reducing the risk of spillover into the human population [7, 8]. For example, oral rabies vaccines that are distributed in bait-form have proven to be effective at controlling rabies in fox and raccoon populations [9–11]. However, even in these cases where an effective bait-deliverable vaccine exists, it remains difficult to achieve a level of vaccination coverage sufficient for pathogen elimination [12, 13]. The key obstacles are the cost and logistical difficulty of distributing vaccine into inaccessible wildlife populations. For zoonotic infectious diseases with short-lived reservoirs (e.g., rodents), the challenge is compounded by the rapid dilution of immunity established through traditional vaccination. These challenges suggest that distributing traditional vaccines as baits is unlikely to provide a general solution [14, 15].

Recent developments in vaccine design offer fresh solutions to this long-standing problem by creating vaccines that are capable of some degree of self-dissemination. Self-disseminating vaccines can be either transferable or transmissible. Development of transferable vaccines has focused on applying topical vaccine-laced gels to individual animals [16]. When other individuals engage in natural allogrooming behaviors common in some reservoir species (e.g., bats), they ingest the vaccine and gain immunity. As a result, the number of animals that can be vaccinated is substantially multiplied [16]. In contrast to transferable vaccines which do not generate sustained chains of self-dissemination, transmissible vaccines are engineered to be contagious, and are potentially capable of indefinite self-dissemination within the reservoir population [17]. A diverse range of modeling studies have demonstrated that both types of self-disseminating vaccines reduce the effort required to achieve herd immunity within wildlife reservoir populations [16–22]. We do not yet know, however, how the introduction of these vaccines can be best timed to maximize their impact when used in reservoir species that have pronounced seasonal population dynamics.

Previous modeling work has demonstrated that the success of traditional wildlife vaccination campaigns can be improved by timing vaccine introduction to coincide with seasonal birth pulses in short-lived animal species [23]. Although intuition suggests similar results should hold for self-disseminating vaccines, the quantitative details remain unknown and important questions remain unanswered. For instance, is timing vaccine introduction more important in transferable vaccines than transmissible vaccines? Do the detailed transmission dynamics of the vaccine (e.g., transmission rate and duration of self-dissemination) influence the optimal timing of introduction? Does timing matter more for some reservoir species than others? Here we develop a general mathematical modeling framework for transmissible and transferable vaccines and use it to quantify the consequences of introducing self-disseminating vaccines at different times throughout the year. We then apply our model to two specific reservoir species that harbor important human pathogens: the primary reservoir of Lassa virus, *Mastomys natalensis*, more commonly known as the multimammete rat and an important carrier of rabies virus, *Desmodus rotundus*, frequently referred to as the common vampire bat. The specific questions we address are: 1) What is the optimal time of year to distribute a self-disseminating vaccine? 2) In which situations is optimal timing critical for success? 3) How does the duration of self-dissemination affect the optimal vaccination strategy? 4) How does host demography influence the importance of timing vaccine distribution?

## Methods

### General methods

We use an SIR (Susceptible-Infected-Recovered) modeling framework to study how the timing of vaccination influences the ability of a self-disseminating vaccine to protect a population from a pathogen. We focus our efforts on populations that undergo seasonal fluctuations in population density driven by well-defined seasonal patterns of reproduction. Our models assume vaccines are introduced into relatively small geographic areas within which the reservoir population is well mixed and of modest size (e.g., 2000 individuals). Thus, our models will apply best to animals that do not live in isolation or in exclusively family groups. These assumptions are motivated by rodent species such as *Mastomys natalensis* and *Peromyscus maniculatus* that harbor important human pathogens such as Lassa virus and Sin Nombre virus, respectively [24, 25].

In the model, we use a time-dependent birth function that is a variation of the periodic Gaussian function developed by [26]:

$$\begin{array}{c} b\left(t\right)=k\cdot e^{-s\cdot {\text{cos}}^{2}\left(\frac{\pi }{365}\cdot t\right)} \end{array}$$ (1)

where *s* tunes the synchrony of births, *k* is set so that the average annual population size is equal to $\bar{N}$, and time is measured in units of days (see S1 Appendix for more details).

For our models, we assume vaccines are 100% efficacious and that direct vaccination occurs each year beginning *t*_*v*_ days after the start of the reproductive season and continues for *V*_*l*_ days. Assuming *N*_*v*_ vaccines are distributed each year (transmissible vaccine) or *N*_*v*_ animals are painted with vaccine-laced gel (transferable vaccine) at a rate *σ*(*t*), the rate at which individuals are directly vaccinated is given by:

$$\begin{array}{c} \sigma \left(t\right)=\{\begin{array}{cc} \frac{N_{v}}{V_{l}} & t_{v}\leq \text{mod}\left(t,365\right)<t_{v}+V_{l}\\ 0 & \text{Otherwise} \end{array}. \end{array}$$ (2)

#### Transmissible vaccine model

Our transmissible vaccine model contains four classes: individuals that are susceptible to both the pathogen and the vaccine (*S*), individuals that are infected with the pathogen (*P*), vaccinated individuals that are immune to the pathogen and capable of transmitting vaccine to susceptible individuals (*V*), and individuals that have immunity due to recovery from pathogen infection or from vaccination (*R*). For simplicity, we assume individuals that have recovered from either the pathogen or the vaccine maintain lifelong immunity to both, and that co-infection with vaccine and pathogen does not occur. Individuals that are infected with the pathogen recover at rate *γ*_*P*_, and individuals infected with the vaccine recover at rate *γ*_*V*_. We assume density-dependent transmission of the pathogen and the vaccine, with transmission coefficients *β*_*P*_ and *β*_*V*_ respectively. Individuals may also be lost from the system due to pathogen-induced mortality at rate *v* or natural mortality at rate *d*. Setting the transmission rate of the vaccine *β*_*V*_ equal to zero yields a model for a traditional vaccination campaign.

**Fig 1:**
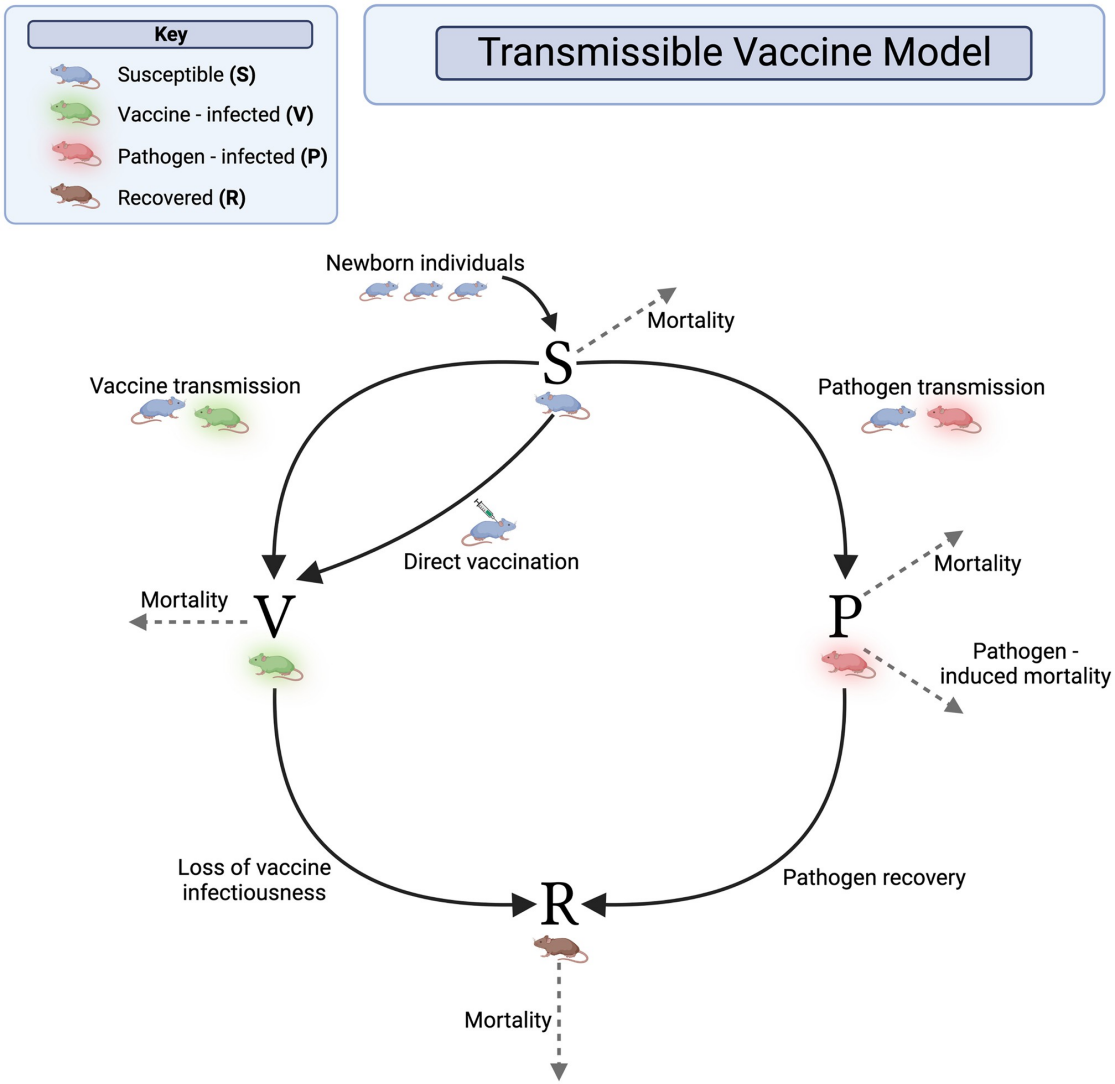
Transmissible vaccine model diagram. Graphical representation of the general framework for the transmissible vaccine model. The model consists of Susceptible individuals (*S*), Pathogen-infected individuals (*P*), Vaccine-infected individuals (*V*), and Recovered individuals (*R*). Individuals enter the system through birth into the susceptible class at the seasonally variable rate *b*(*t*) (Eq 1), all individuals leave the system through disease-independent mortality (*d*) and infected individuals may experience additional disease-dependent mortality at rate (*ν*). Other important parameters are the transmission rate of the pathogen (*β*_*P*_), recovery from the pathogen (*γ*_*P*_), direct-vaccination $\sigma \left(t\right)\frac{S}{N}$ (Eq 2), transmission of the vaccine (*β*_*V*_), and loss of vaccine transmissibility (*γ*_*V*_). Further details of the model can be found in the main text and the full system of equations can be found in the S1 Appendix. These figures were created with BioRender.com.

Susceptible individuals can be vaccinated directly or by coming into contact with vaccine-infected individuals. Because we assume vaccines are administered to animals at random, vaccines will be applied to any individual in the population, including individuals already immune to the pathogen, such that at least some waste is inevitable. We model this feature of vaccine distribution by multiplying the rate at which vaccines are deployed at time t, *σ*(*t*), by the fraction of susceptible individuals $\left(\frac{S}{N}\right)$ in the population. Here, *N* denotes the total population size. Thus, if the entire population is susceptible, vaccination efficiency is high and waste is low. In contrast, if the population contains a large proportion of immune individuals, vaccination efficiency is low and waste is high. A description of all model parameters can be found in Table 1, and a graphical representation of the model is given in Fig 1. The full set of model equations can be found in the S1 Appendix.

**Table 1:** Table of model parameters and biological interpretation.

Parameter	Description
*t*_*v*_	Day in year of vaccine initiation
*V*_*l*_	Duration of the vaccination campaign (days)
*s*	Synchrony of births
*d*	Natural mortality rate (per individual per day)
$\bar{N}$	Average population size
*R*_0,*V*_	*R*_0_ of the vaccine
*R*_0,*P*_	*R*_0_ of the pathogen
*γ*_*P*_	Recovery rate of the pathogen (per individual per day)
*γ*_*V*_	Recovery rate of the transmissible vaccine (per individual per day)
*γ*_*g*_	Recovery rate of the transferable vaccine (per individual per day)
*β*_*P*_	Rate of pathogen transmission (per individual per day)
*β*_*V*_	Rate of transmissible vaccine transmission (per individual per day)
*β*_*g*_	Rate of transferable vaccine transmission (per individual per day)
*ν*	Rate of pathogen induced mortality (per individual per day)
*α*	Rate at which individuals remove gel via grooming (per individual per day)

#### Transferable vaccine model

Our transferable vaccine model contains five classes: individuals that are susceptible to the pathogen (*S*), individuals that are currently infected by the pathogen (*P*), individuals that are immune to the pathogen (*R*), individuals that are currently infected by the pathogen and also carrying the vaccine-laced topical gel (*P*_*g*_), and individuals that are immune to the pathogen and also carrying the vaccine laced topical gel (*R*_*g*_). We assume vaccine-laced gel is applied topically to captured animals at rate *σ*(*t*). These animals are also assumed to be directly vaccinated upon capture so that susceptible individuals immediately transition to the *R*_*g*_ class. In contrast to the transmissible vaccine model, the rate of vaccination is multiplied by $\frac{1}{S+P+R}$ rather than $\frac{1}{N}$. This is because we assume that if individuals have gel on them, it will be recognized and additional gel will not be applied and wasted. Allogrooming behavior allows an individual to become vaccinated at rate *β*_*g*_ if it encounters an individual carrying the vaccine-laced gel. At the same time, however, allogrooming behavior also depletes the quantity of vaccine-laced gel on individual carriers. We model this phenomenon by assuming the topical gel is lost at rate *αN* which implies gel is lost more rapidly in densely populated animal populations. Additionally, we assume the topical gel loses its ability to serve as a vaccine over time at rate *γ*_*g*_.

**Fig 2:**
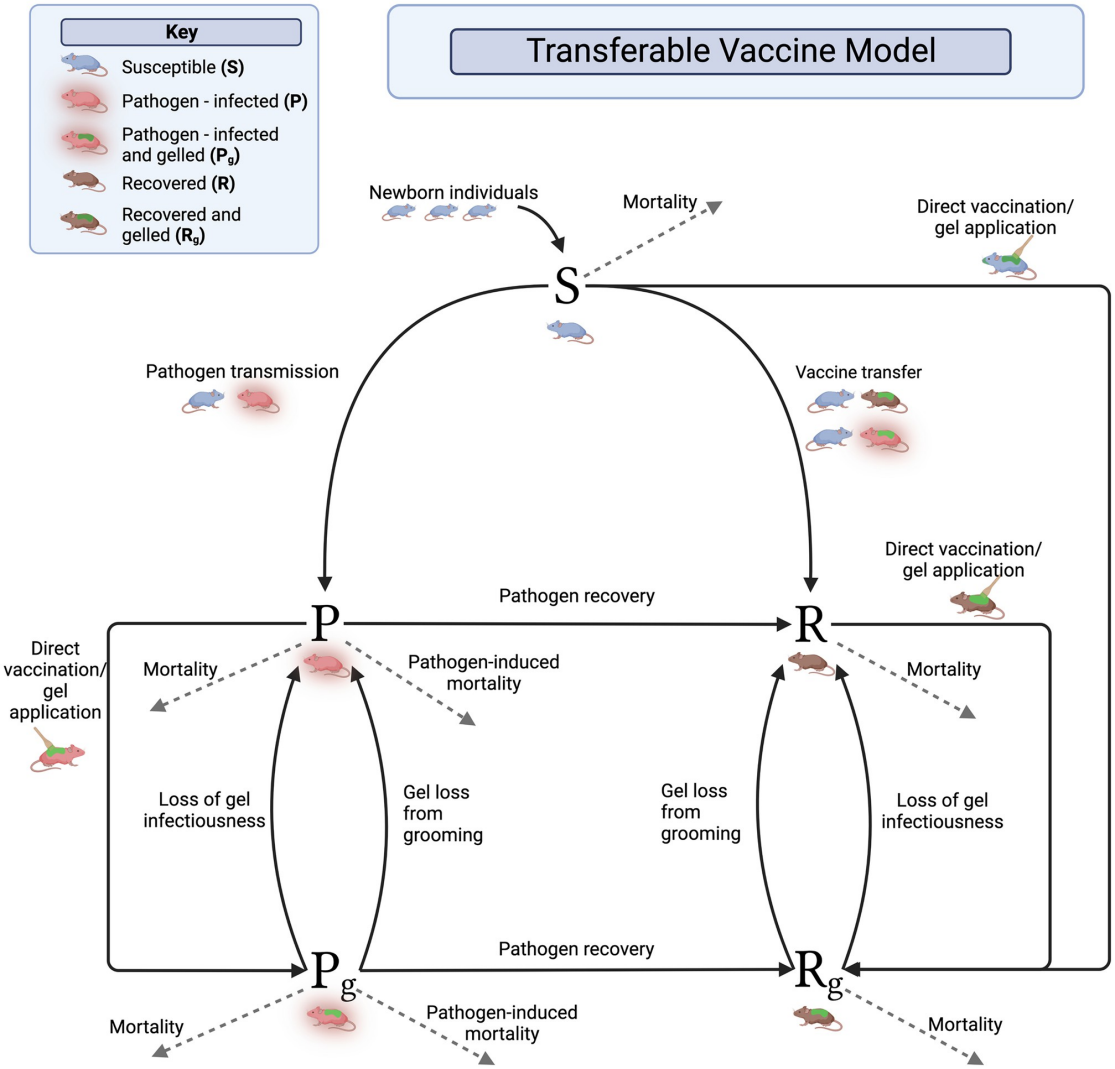
Transferable vaccine model diagram. Graphical representation of the general framework for the transferable vaccine model. The model consists of Susceptible individuals (*S*), Pathogen-infected individuals (*P*), and Recovered individuals (*R*). In addition, we track individuals that have gel on them, that is, Pathogen-infected individuals with gel (*P*_*g*_) and Recovered individuals with gel (*R*_*g*_). There is no Susceptible class with gel because we assume animals to which gel is applied are also directly vaccinated and thus move directly to the recovered and gelled class (rightmost arrow). Individuals are born into the Susceptible class at a seasonally variable rate *b*(*t*) (Eq 1) and all individuals leave the system through disease-independent mortality (*d*) and infected individuals may experience additional disease-dependent mortality at rate (*ν*). Other important parameters are the transmission rate of the pathogen (*β*_*P*_), recovery from the pathogen (*γ*_*P*_), direct-vaccination $\sigma \left(t\right)\frac{S}{S+P+R}$ (Eq 2), transmission of the vaccine-laced gel (*β*_*g*_), deterioration of gel (*γ*_*g*_), and removal of gel via grooming behaviors (*α*). Further details of the model can be found in the main text and the full system of equations can be found in the S1 Appendix. These figures were created with BioRender.com.

We assume that transfer of the vaccine can occur only from an individual to which vaccine-laced gel has been directly applied and that vaccine transfer is density-dependent. Pathogen transmission is also assumed to be density-dependent and to occur at rate *β*_*P*_ from contact with either a pathogen-infected individual (*P*) or a gelled and pathogen-infected individual (*P*_*g*_). See Table 1 for parameter descriptions. A graphical representation of the model can be found in Fig 2 and the full set of equations can be found in the S1 Appendix.

#### Assessment of vaccination strategy

We evaluate the success of a vaccination campaign by comparing the reduction of pathogen-infected individuals it achieves relative to the situation where no vaccination occurs. For each type of vaccine and distribution strategy, we use the deSolve package in R to numerically solve the corresponding system of differential equations [27]. For each combination of parameters we solve the system of differential equations twice: once with vaccination and once without vaccination. Initial conditions are identical for these two cases and both are simulated for 100 years, allowing the system to settle into stable seasonal cycles (burn-in period). One numerical solution is continued from this point for ten years with no vaccination occurring and the other is run with vaccination occurring *t*_*v*_ days into the year, every year, for ten years after the first day of vaccination. We then extract from each of the numerical solutions the average number of pathogen-infected hosts over the ten-year period following the burn-in. Specifically, we calculate the fractional reduction of pathogen-infected individuals (average level of pathogen reduction) provided by vaccination as:

$$\begin{array}{c} \frac{{\bar{x}}_{0}-{\bar{x}}_{v}}{{\bar{x}}_{0}} \end{array}$$ (3)

where ${\bar{x}}_{0}$ is the average number of pathogen-infected individuals in the scenario without vaccination and ${\bar{x}}_{v}$ is the average number of pathogen-infected individuals with vaccination. We use this comparative approach to explore how the benefits of vaccination change as a function of vaccine properties, reservoir properties, and the timing of vaccine introduction. Additionally, we use the concept of the basic reproductive number, denoted as *R*_0_, to compare the relative transmissibility of the vaccine and the pathogen. *R*_0_ represents the average number of new infections caused by a single infected individual that is introduced into a fully susceptible population [28]. More details on the *R*_0_ calculations for transmissible and transferable vaccines can be found in the S1 Appendix.

### Case studies

Up to this point we have developed general models to explore a wide range of parameter space. Our goal was to develop a general understanding of the performance of self-disseminating vaccines as a function of reservoir biology, vaccine properties, and introduction protocol. Next, we shift our focus to specific hosts and the pathogens they carry. We use estimates from the literature to parameterize our model and draw conclusions for two specific systems where self-disseminating vaccines are being developed. Specifically, we focus on the primary rodent reservoir of Lassa virus, *Mastomys natalensis* and a bat reservoir of rabies virus *Desmodus rotundus*. A table of parameter values used in both the general simulations and specific case studies can be found in the S1 Table. In addition, the full systems of differential equations for each case study can be found in the S1 Appendix.

#### *Mastomys natalensis*—Lassa virus

Our first case-study is the primary rodent reservoir of Lassa virus, *M. natalensis*. Lassa virus commonly spills over into the human population through contact with rodent urine or feces and can lead to the development of Lassa fever which can be fatal in humans [29, 30]. The modeling framework used for the *M. natalensis* case study is the same as that described above for the general model (Eq 4 and Eq 5 in the S1 Appendix). Population sizes of *M. natalensis*, have been shown to fluctuate seasonally in response to birth pulses coinciding with the beginning of the wet season and an increase in the availability of green grass as well as other food sources [29, 31]. We use data from a study in Guinea—where Lassa virus is endemic—to estimate the level of seasonality that these populations demonstrate [32]. Here, we set demographic parameters that fix the average population size to 2000 individuals, as estimated by [15]. Additionally, parameters estimated from [14] suggest a lifespan of one year for the rodent reservoir, a rate of recovery from Lassa virus infection equal to 21 days, and a Lassa virus *R*_0,*P*_ = 1.5. We are then able to solve for the transmission coefficient *β*_*P*_ based on *γ*_*p*_ and *R*_0,*P*_ (See S1 Appendix). We base the transmissible vaccine parameters on a recent study [33] which assumes that the rodents would be infectious with the vaccine for their entire life (*γ*_*v*_ = 0). We consider a range of values for the reproductive number of the vaccine (*R*_0,*V*_) and we use this predefined *R*_0,*V*_ as well as the recovery rate to calculate the transmission rate of the vaccine (See S1 Appendix).

#### *Desmodus rotundus*—rabies virus

Our second case-study focuses on the vampire bat, *D. rotundus*, which serves as a reservoir for rabies virus within Central and South America. Rabies is a disease caused by *Rabies lyssavirus* commonly spread by bats and is fatal in most mammals, including humans [34]. The modeling framework used for the *D. rotundus* case study is the same as that described above for the general model (Eq 4 and Eq 5 in the S1 Appendix). However, instead of assuming density-dependent transmission, we assume frequency-dependent transmission for both the pathogen and self-disseminating vaccines which has been well established for this system (See Eq 6 and Eq 7 in the S1 Appendix for the complete model.) [16, 35–37]. Vampire bats show evidence of seasonal births and previous studies have used lactation rates to estimate the reproductive seasonality in these populations [36]. We tailor our birth function to data on lactation from [38] (See S1 Appendix). Although local population sizes of *D. rotundus* are unclear, estimates for colony size do exist. For this reason we focus on a vaccination campaign targeting a single colony of 240 individuals as estimated by [16]. Estimates suggest that *D. rotundus* live for an average of three and a half years [39]. To simulate the pathogen dynamics of rabies we use a pathogen *R*_0_ of 1.5 and an average duration of infection of 21 days [36, 40, 41]. We assume individuals infected with the pathogen have a 10% chance of dying due to infection [16, 36].

Because both transferable and transmissible vaccines are currently being developed for *D. rotundus* we study both scenarios. Specifically, we assume the transferable vaccine gel stays on for approximately two days (*γ*_*g*_ = 1/2) as suggested by [16]. Because the proposed vector for a rabies virus transmissible vaccine is a betaherpesvirus, we assume the vaccine will induce lifelong infection (*γ*_*v*_ = 0) [37, 42]. Recent work from [37] estimated the *R*_0_ for a promising betaherpesvirus vector to be 6.9 in this system. Because we anticipate that engineering any viral vector to carry an immunogenic insert will reduce its transmissibility, we considered *R*_0_ values considerably below this estimate for the wild type vector.

## Results

### General results

#### Temporal dynamics of immunity depend on the type of self-disseminating vaccine

**Fig 3:**
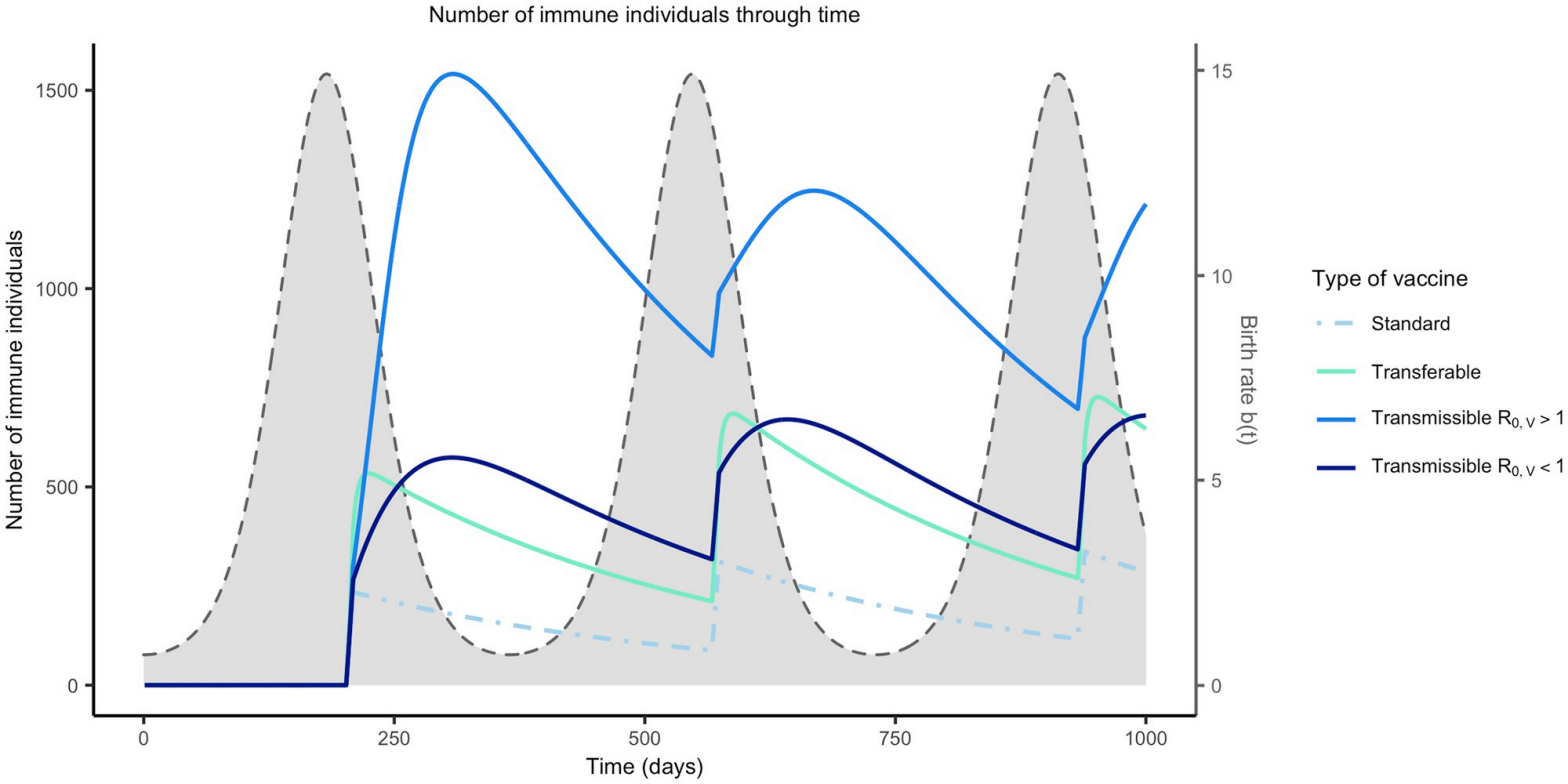
Temporal dynamics of immunity. The temporal dynamics of immunity for standard, transferable, and transmissible vaccines in the absence of a pathogen. For each type of vaccine, 250 vaccines are distributed on day 200. The colored lines represent the number of immune individuals in the population over three years of repeated vaccination for either a standard vaccine, transferable vaccine, transmissible vaccine with *R*_0,*V*_ < 1, and a transmissible vaccine with *R*_0,*V*_ > 1. *R*_0,*V*_ of the standard, transferable, strongly transmissible, and weakly transmissible are: (0, 1.5, 1.5, and 0.75) respectively. The remaining parameters are: an average population size of 2000 individuals ($\bar{N}=2000$), *s* = 3, an average lifespan of 1 year (*d* = 1/365), *R*_0,*P*_ = 2, 250 vaccines are distributed each year (*N*_*V*_ = 250), individuals can disseminate vaccine for 21 days on average (*γ*_*V*_ = 21^−1^), individuals remain infectious with the pathogen for 21 days on average (*γ*_*P*_ = 21^−1^), the transferable vaccine is groomed off individuals after 6 days on average (*α* = 1/15000, and the pathogen is non-virulent (*ν* = 0).

Previous work has demonstrated that self-dissemination increases vaccine coverage and reduces the effort required for pathogen elimination [17]. However, it remains unclear how self-disseminating vaccines will perform in fluctuating populations. To establish baseline expectations for the performance of self-disseminating vaccines in fluctuating reservoir populations we begin by studying the dynamics of immunity in the absence of the pathogen. Numerical analyses performed over a wide range of parameters demonstrate that the temporal dynamics of immunity differ across vaccine types in characteristic ways (Fig 3). For conventional vaccines that lack the ability to self-disseminate, vaccination results in a rapid increase in the number of vaccinated individuals, followed by a decrease due to the continued influx of susceptible individuals during the birthing season. Transferable vaccines result in similar temporal dynamics but show a transient increase in immunity from self-dissemination following vaccine introduction. In contrast, transmissible vaccines with an *R*_0,*V*_ > 1 can continue to increase the number of immune individuals long after vaccine introduction because they generate self-sustaining chains of transmission. Because all individuals die at a constant rate *d*, the number of immune individuals decreases after the birth pulse ends until the next vaccination campaign for all types of vaccine. With self-disseminating vaccines, the level of increase in the number of immune individuals in the population is dependent on the vaccine *R*_0_ (*R*_0,*V*_) (Fig 3).

#### Timing is critical for most self-disseminating vaccines

**Fig 4:**
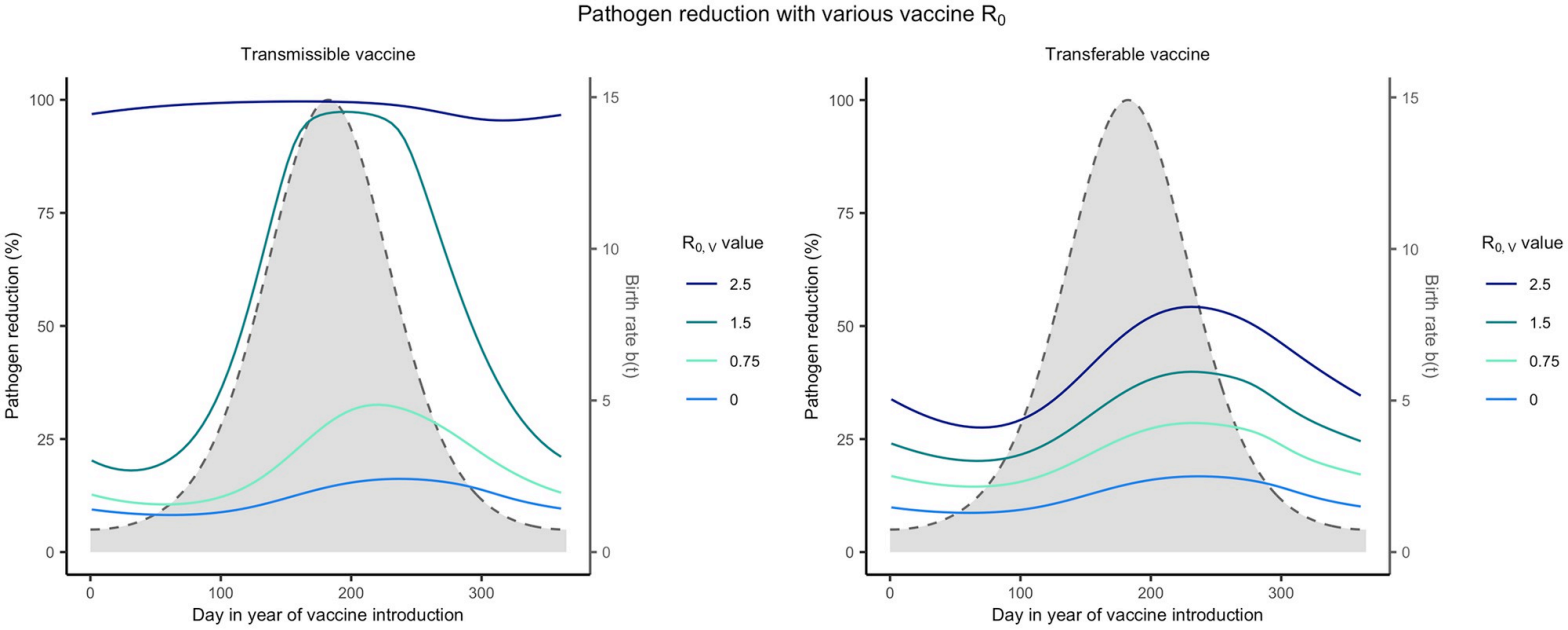
R_0,V_ and the importance of timing vaccine delivery. Optimal timing for self-disseminating vaccines as a function of vaccine *R*_0,*V*_. Solid lines represent the level of pathogen reduction achieved for a given date of vaccine introduction for different vaccine *R*_0,*V*_. The grey region outlined by the dashed lined represents the seasonal birthing season where day 1 corresponds to the first day of the birthing season. Additional parameters used were: an average population size of 2000 individuals ($\bar{N}=2000$), *s* = 3, an average lifespan of 1 year (*d* = 1/365), *R*_0,*P*_ = 2, 250 vaccines are distributed each year (*N*_*V*_ = 250), individuals can disseminate vaccine for 21 days on average (*γ*_*V*_ and *γ*_*g*_ = 21^−1^), individuals remain infectious with the pathogen for 21 days on average (*γ*_*P*_ = 21^−1^), the transferable vaccine is groomed off individuals after 6 days on average (*α* = 1/15000, and the pathogen is non-virulent (*ν* = 0).

Previous work has shown that the timing of delivery for conventional vaccines matters in short-lived animals with distinct reproductive seasons [23]. Here, our goal is to evaluate whether timing is more important for transmissible or transferable vaccines and under which conditions timing matters most. To this end, we compared the reduction in pathogen prevalence achieved for vaccination campaigns that are initiated at different times of year and last for various lengths of time. Our results demonstrate that distributing self-disseminating vaccines slightly after the peak of the birthing season will substantially reduce pathogen prevalence (Fig 4). This occurs because it is at this time that population density and the proportion of susceptible individuals are near their seasonal maxima. This ensures that vaccines are not wasted by distributing vaccine at the wrong time. If, however, a large number of vaccines are available and can be distributed, a greater level of pathogen reduction can be achieved and the importance of timing decreases (Fig A in S1 Text).

For both types of self-disseminating vaccine, pathogen reduction is greater with a larger vaccine *R*_0_. In addition to facilitating pathogen elimination, increasing the transmissible vaccine’s *R*_0,*V*_ also increases the range of times over which a vaccine can be introduced and still substantially reduce the pathogen’s prevalence (Fig 4). This occurs because increased transmission allows the vaccine to be introduced earlier in the reproductive season and still reach individuals that will be born later through downstream transmission. In contrast, with reduced transmission (lower *R*_0,*V*_), if a transmissible vaccine is introduced too early, chains of transmission are generally too short to reach individuals born later in the season resulting in wasted vaccine. Once the *R*_0,*V*_ of the transmissible vaccine exceeds that of the pathogen *R*_0,*P*_, timing matters little and significant pathogen reductions can be accomplished for a broad range of introduction times (Fig 4). This is because a vaccine more transmissible than the target pathogen can out-compete the pathogen and will inevitably displace it from the population over time [18]. A fundamental difference for transferable vaccines is that they never reach this same level of insensitivity to the timing of introduction. The reason for this is that they are (by definition) capable of spreading only from individuals that have been directly vaccinated and thus generate chains of transmission only one step long. Because of this limited spread, an increased *R*_0,*V*_ of the transferable vaccine results in higher levels of pathogen reduction, but not an increase in the range of times over which high pathogen reduction can be achieved (Fig 4).

In general, self-disseminating vaccines should be distributed after the peak of the birthing season to maximize their impact. Specifically, transferable vaccines cause the greatest reduction in the number of pathogen-infected individuals when introduced after the peak of the birthing season. In contrast, transmissible vaccines cause the greatest reduction in the number of pathogen-infected individuals when introduced during the birth pulse, with the optimal solution depending on vaccine *R*_0_. Specifically, the impact of transmissible vaccines with intermediate *R*_0,*V*_ is maximized by early introduction. This occurs because these highly transmissible vaccines can be introduced when newly born susceptible individuals are relatively rare and yet still reach susceptible individuals born later. In contrast, transmissible vaccines with small *R*_0,*V*_ must be introduced later and after a significant number of susceptible individuals has accumulated in order to persist and spread (Fig 4).

**Fig 5:**
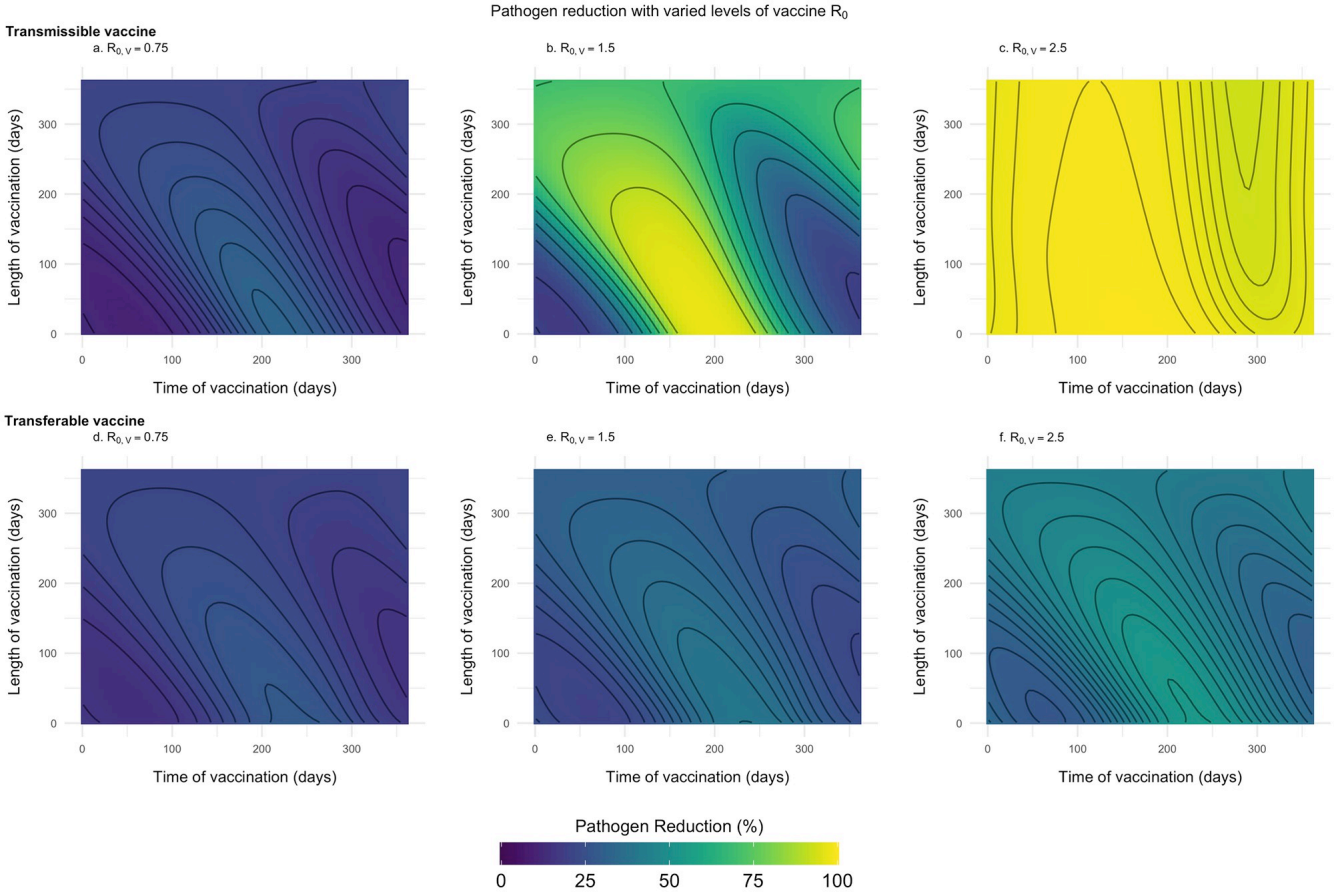
Level of pathogen reduction across various R_0,V_, t_v_, and V_l_. Level of pathogen reduction achieved for both transmissible vaccines and transferable vaccines at different times (*t*_*v*_) and for different durations of a vaccination campaign (*V*_*l*_). The *R*_0_ in the figure refers to the vaccine *R*_0_. The remaining parameters used were: an average population size of 2000 individuals ($\bar{N}=2000$), *s* = 3, an average lifespan of 1 year (*d* = 1/365), *R*_0,*P*_ = 2, 250 vaccines are distributed each year (*N*_*V*_ = 250), individuals can disseminate vaccine for 21 days on average (*γ*_*V*_ and *γ*_*g*_ = 21^−1^), individuals remain infectious with the pathogen for 21 days on average (*γ*_*P*_ = 21^−1^), the transferable vaccine is groomed off individuals after 6 days on average (*α* = 1/15000, and the pathogen is non-virulent (*ν* = 0).

For vaccination campaigns of feasible duration (one week—2 months), the duration of the vaccination campaign itself matters little as long as the total amount of distributed vaccine is held fixed (Fig 5). This insensitivity arises primarily because birth rates change little over such short periods of time in most systems. Vaccination campaigns with the same number of vaccines but longer durations lead to lower levels of pathogen reduction because vaccines are distributed when fewer susceptible individuals exist within the reservoir population. If, however, the vaccination campaign begins at the wrong time (i.e., before or after the birthing season), extending the duration of vaccine-delivery can increase pathogen reduction by reducing the number of vaccines that are distributed at inopportune times (Fig 5). If the timing of birthing within the reservoir population is known, however, the best solution for maximizing the reduction in pathogen prevalence is to distribute vaccines shortly after the peak of the birthing season and over a relatively short amount of time.

#### Vaccines with temporally focused self-dissemination are more effective

Because vaccines may differ widely in the period of time over which they self-disseminate, we explored how this property influenced the optimal timing of delivery. For both types of vaccines, we considered scenarios where the vaccine remained infectious for 14, 21, 30, 182, and 365 days on average, with vaccine *R*_0,*V*_ held constant at a value of 1.5. Holding *R*_0,*V*_ constant while changing the duration of self-dissemination (infectious period − 1/*γ*_*V*_) requires that the rate of vaccine transmission also changes *β*_*V*_. Thus, vaccines with temporally focused periods of self-dissemination also have a high transmission rate whereas vaccines with drawn out periods of self-dissemination have a low transmission rate. If, however, the vaccine *R*_0_ is not held constant by changing the rate of vaccine transmission, then increasing the duration of self dissemination increases vaccine *R*_0,*V*_ leading to higher levels of pathogen reduction.

**Fig 6:**
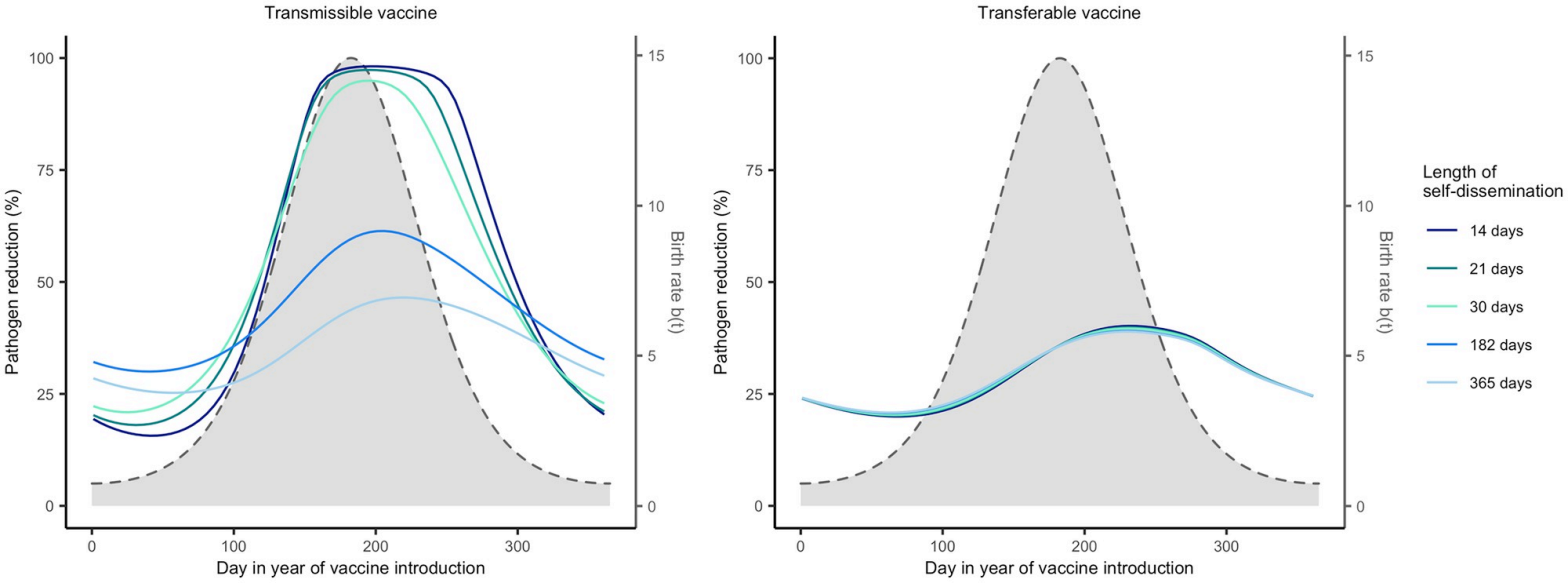
Pathogen reduction across different vaccine infectious periods. Level of pathogen reduction achieved across various times of vaccination with different vaccine recovery rates indicated by the different colors. The vaccine recovery rate controls the length of time that the vaccine can disseminate to other individuals in the population. Solid lines represent the level of pathogen reduction achieved for a given date of vaccine introduction. The grey region outlined by the dashed line represents the birthing season where day 1 corresponds to the first day of the birthing season. The remaining parameters are: an average population size of 2000 individuals ($\bar{N}=2000$), *s* = 3, an average lifespan of 1 year (*d* = 1/365), *R*_0,*V*_ = 1.5, *R*_0,*P*_ = 2, 250 vaccines are distributed each year (*N*_*V*_ = 250), individuals remain infectious with the pathogen for 21 days on average (*γ*_*P*_ = 21^−1^), the transferable vaccine is groomed off individuals after 6 days on average (*α* = 1/15000, and the pathogen is non-virulent (*ν* = 0).

Our results indicate that vaccines that disseminate for short periods of time are more effective and create greater opportunity for pathogen reduction (Fig 6). This result hinges on our assumption of a fixed *R*_0_ of the vaccine. With a fixed *R*_0,*V*_, lowering the duration of infection leads to higher rates of transmission. In contrast, increasing the duration of infection leads to lower rates of transmission. Vaccines that have short infectious periods spread more rapidly and lead to greater pathogen reduction if distributed at the optimal time. In contrast, vaccines that that have long infectious periods spread more slowly and thus lead to lower levels of pathogen reduction when distributed at the optimal time. However, it is important to note that since the transferable vaccine is groomed off of individuals at rate (*α*), vaccines with infectious periods that are longer than the average duration gel remains on individuals show no difference (Fig 6). The reverse is also found if we compare different alpha values (Fig B in S1 Text). Overall, we find that although the duration of self-dissemination influences the effectiveness of self-disseminating vaccines, it has little impact on the optimal timing of vaccine introduction: it is generally best to distribute the transmissible vaccine during the birthing season and the transferable vaccine slightly after the peak of the birthing season. Similarly, the duration of the infectious period for the pathogen has little affect on the optimal timing of vaccine delivery, although longer infectious periods decrease the vaccines ability to reduce pathogen prevalence (Fig C in S1 Text).

#### Reservoir life history modulates the importance of vaccine timing

**Fig 7:**
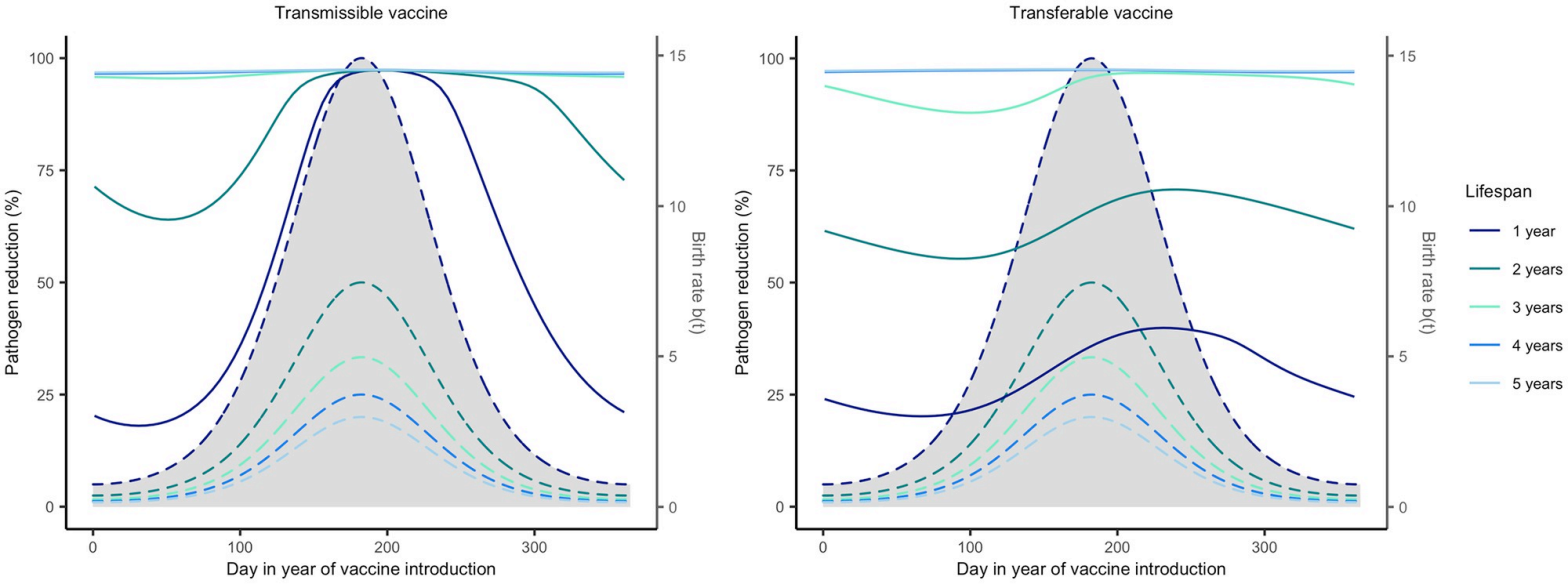
Pathogen reduction across different host lifespans. Level of pathogen reduction achieved across various times of vaccination with different average host lifespans indicated by the different colors. Solid lines represent the level of pathogen reduction achieved for a given date of vaccine introduction. Dashed lines and the grey region beneath them represent the birthing season. Day 1 corresponds to the first day of the birthing season as well as the first possible day of vaccine introduction. The remaining parameters are: an average population size of 2000 individuals ($\bar{N}=2000$), *s* = 3, *R*_0,*V*_ = 1.5, *R*_0,*P*_ = 2, 250 vaccines are distributed each year (*N*_*V*_ = 250), individuals can disseminate vaccine for 21 days on average (*γ*_*V*_ and *γ*_*g*_ = 21^−1^), individuals remain infectious with the pathogen for 21 days on average (*γ*_*P*_ = 21^−1^), the transferable vaccine is groomed off individuals after 6 days on average (*α* = 1/15000, and the pathogen is non-virulent (*ν* = 0).

**Fig 8:**
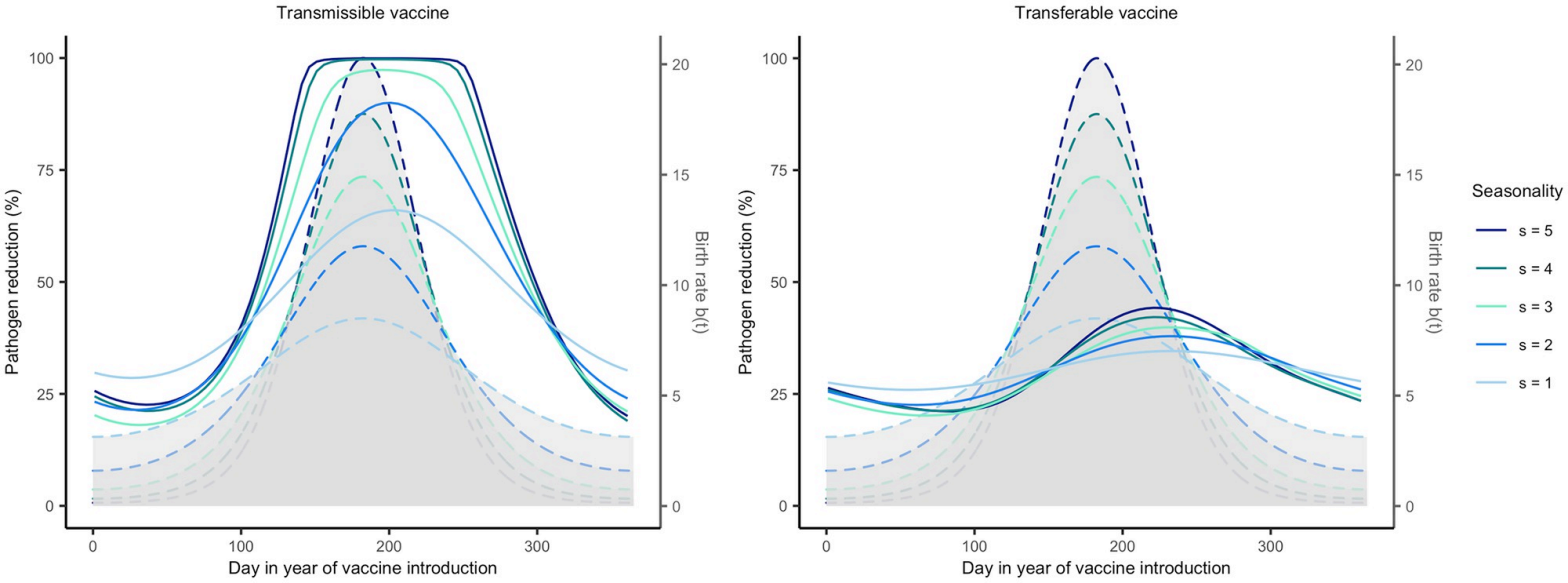
Pathogen reduction across differing levels of host seasonality. Level of pathogen reduction achieved across various times of vaccination for varying levels of synchronous births (*s*). Low *s* or low synchrony implies births occur over a large amount of time whereas high s or high synchrony implies all births occur over a very short time frame. Solid lines represent the level of pathogen reduction achieved for a given date of vaccine introduction. The grey region outlined by the dashed colored lines represent the birthing season for the respective parameter regime shared with the solid lines. Day 1 corresponds to the first day of the birthing season as well as the first possible day of vaccine introduction. The remaining parameters are: an average population size of 2000 individuals ($\bar{N}=2000$), an average lifespan of 1 year (*d* = 1/365), *R*_0,*V*_ = 1.5, *R*_0,*P*_ = 2, 250 vaccines are distributed each year (*N*_*V*_ = 250), individuals can disseminate vaccine for 21 days on average (*γ*_*V*_ and *γ*_*g*_ = 21^−1^), individuals remain infectious with the pathogen for 21 days on average (*γ*_*P*_ = 21^−1^), the transferable vaccine is groomed off individuals after 6 days on average (*α* = 1/15000, and the pathogen is non-virulent (*ν* = 0).

We investigated how reservoir life history influences the importance of vaccine timing by adjusting average lifespan and the seasonality of reproduction. Our results demonstrate that the importance of vaccine timing decreases as average lifespan increases and has little impact when average lifespan exceeds 3 years (Fig 7). This occurs because long-lived reservoir species have a reduced rate of population turnover such that immune individuals persist within the population rather than being replaced by large quantities of susceptible individuals during the seasonal birth pulse. Even among hosts with highly synchronous births, but long lifespans, timing the delivery of vaccine made little difference in the level of pathogen reduction achieved due to long lived hosts having overall lower birth rates (Fig E in S1 Text). For those hosts with relatively brief lifespans (e.g., < 3 years), seasonality increases the importance of timing and the effectiveness of the vaccination campaign (Fig 8). This occurs because reproductive seasonality concentrates births and creates periods of time where large numbers of susceptible individuals circulate within the reservoir population. This creates opportunities for a self-disseminating vaccine to spread to a large number of individuals if its introduction is well-timed. This effect is magnified for transmissible vaccines because of their increased potential for self-dissemination.

### Case study results

#### *Mastomys natalensis*—Lassa Virus

**Fig 9:**
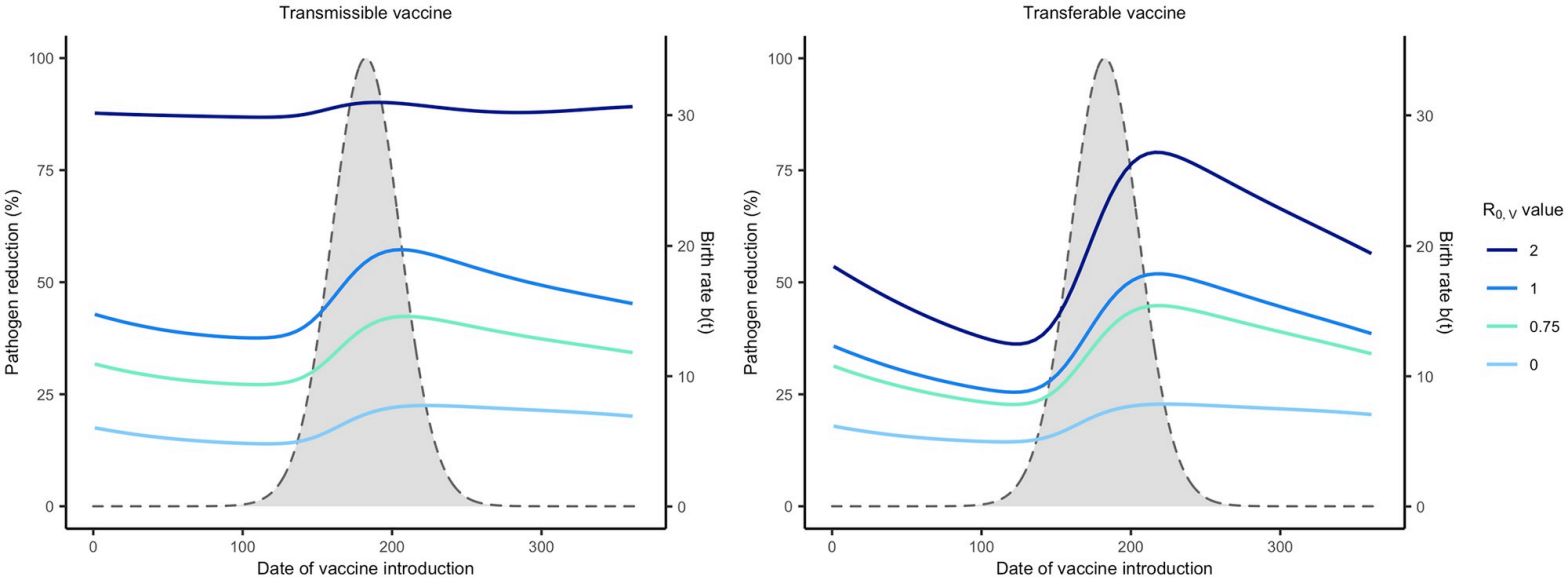
Rodent case study example. Specific example for *M. natalensis* that describes the level of pathogen reduction achieved across various times of vaccination with different vaccine *R*_0_ values indicated by the different colors. Solid lines represent the level of pathogen reduction achieved for a given date of vaccine introduction. The grey region outlined by the dashed line represents the birthing season where day 1 corresponds to the first day of the birthing season. The remaining parameters used were: an average population size of 2000 individuals ($\bar{N}=2000$), *s* = 13.078, an average lifespan of 1 year (*d* = 1/365), *R*_0,*P*_ = 1.5, 200 vaccines are distributed each year (*N*_*V*_ = 200), individuals can disseminate the transferable vaccine for 2 days on average (*γ*_*g*_ = 2^−1^), individuals remain infectious with the transmissible vaccine for their entire life (*γ*_*V*_ = 0), individuals remain infectious with the pathogen for 21 days on average (*γ*_*P*_ = 21^−1^), the transferable vaccine is groomed off individuals after 6 days on average (*α* = 1/15000, and the pathogen is non-virulent (*ν* = 0).

We studied simulated vaccination campaigns of both the transmissible and transferable vaccine targeting Lassa virus in *M. natalensis* using the models and parameters described in the methods section. Lassa virus is estimated to have an *R*_0,*P*_ = 1.5. Our model suggests that a transmissible vaccine with an *R*_0,*V*_ = 1 could achieve a reduction in LASV prevalence of 57% if the vaccine is introduced at the optimal time but only 37.5% if introduced before the birthing season and a transferable vaccine with *R*_0,*V*_ = 1 could achieve a 52% reduction in pathogen prevalence if timed correctly in contrast to a 25% reduction in pathogen prevalence if delivered too early. These simulations demonstrate that Lassa virus prevalence within the reservoir population is maximally reduced when vaccines are introduced shortly after the peak of the birthing season (Fig 9). Although the precise reduction likely to be achieved in real world applications will depend on the intensity of seasonal fluctuations and local ecological conditions, our simulations suggest the conclusions for optimal timing are quite general. These results also assume a recombinant vector transmissible vaccine that causes long-term chronic infections. A transmissible vaccine constructed from a vector that generates short-term acute infections would be even more sensitive to accurate timing.

#### *Desmodus rotundus*—Rabies Virus

**Fig 10:**
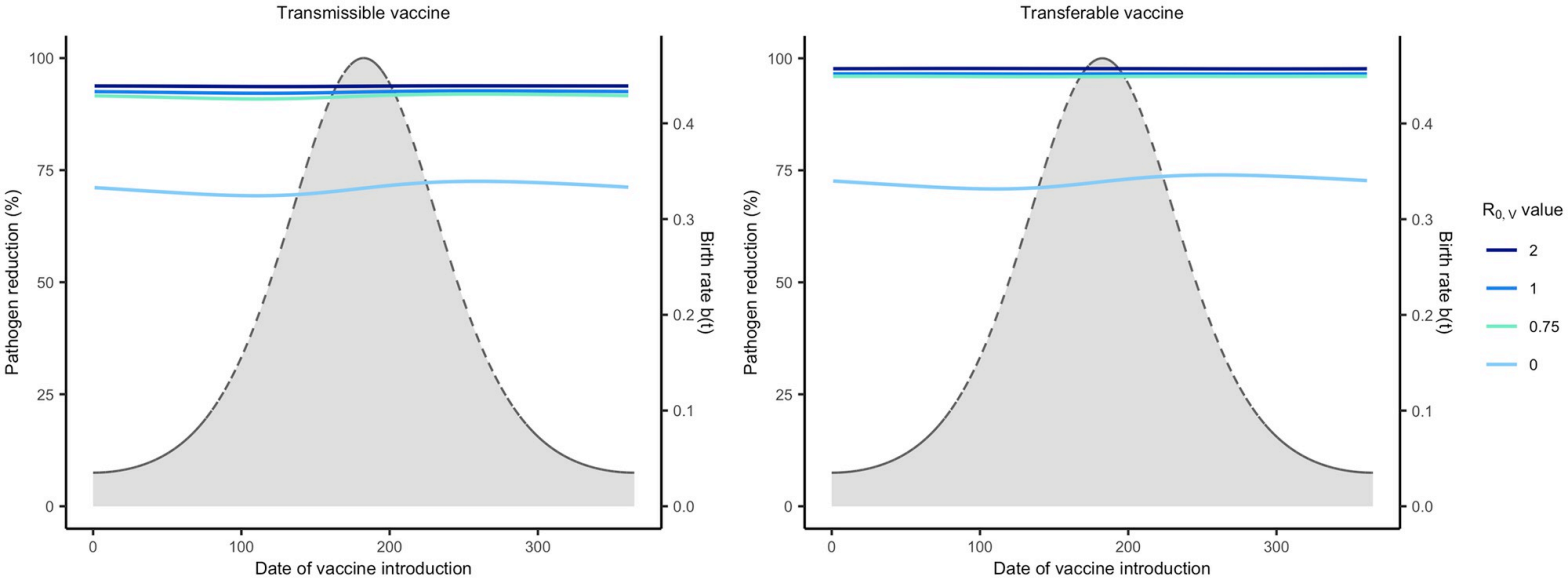
Bat case study example. Specific example for *D. rotundus* on the level of pathogen reduction achieved across various times of vaccination with different vaccine *R*_0_ values indicated by the different colors. Solid lines represent the level of pathogen reduction achieved for a given date of vaccine introduction. The grey region outlined by the dashed lined represents the birthing season where day 1 corresponds to the first day of the birthing season. The remaining parameters used were: an average population size of 240 individuals ($\bar{N}=240$), *s* = 2.59, an average lifespan of 3.5 years (*d* = 1/(365 × 3.5), *R*_0,*P*_ = 1.5, 24 vaccines are distributed each year (*N*_*V*_ = 24), individuals can disseminate the transferable vaccine for 7 days on average (*γ*_*g*_ = 2^−1^), individuals remain infectious with the transmissible vaccine for their entire life (*γ*_*V*_ = 0), individuals remain infectious with the pathogen for 21 days on average (*γ*_*P*_ = 21^−1^), the transferable vaccine is groomed off individuals after 6 days on average (*α* = 1/15000, and the pathogen is virulent (*ν* = 0.005).

In addition to *M. natalensis*, we studied simulated vaccination campaigns using both transmissible and transferable vaccines targeting rabies virus in *D. rotundus*. In contrast to Lassa virus, the models we use to study rabies virus assume transmission is frequency-dependent (Eq 6 and Eq 7 in S1 Appendix). Under this model framework and with vampire bat rabies having an estimated *R*_0,*P*_ = 1.5, our results suggest a transmissible vaccine with an *R*_0,*V*_ = 1 could achieve a 93% reduction in rabies virus prevalence and a transferable vaccine with *R*_0,*V*_ = 1 could achieve a 96.5% reduction in pathogen prevalence. These results are similar to a recent study that models this system more explicitly. [37] found a betaherpesvirus-vectored vaccine could achieve a 60%—94% reduction in outbreak size depending on vaccine *R*_0_ and vaccine coverage. The transferable vaccine achieves a higher level of pathogen reduction here due to the shorter duration of self-dissemination (*γ*_*g*_ = 2^−1^), where as the transmissible vaccine causes lifelong infection of the vaccine (*γ*_*V*_ = 0). As seen previously in Fig 6, longer durations of self-dissemination lead to lower levels of pathogen reduction because—when *R*_0,*V*_ is held constant—the vaccine must have a lower transmission rate. Our simulations demonstrate that both types of self-disseminating vaccines could substantially reduce viral prevalence within the bat population regardless of when they are distributed relative to the birthing season (Fig 10). Although our simulation results suggest our conclusions about timing are robust, the precise reductions in pathogen prevalence that are achieved will vary depending on local ecological conditions, the intensity of seasonal fluctuations, and vaccine design. The large reductions in pathogen prevalence and the insensitivity to timing of vaccine delivery seen here for both types of vaccine are due to the substantially longer lifespan of *D. rotundus* compared to *M. natalensis*. As discussed above, organisms with longer lifespans are less sensitive to timing because these populations have low influxes of susceptible individuals each year. In contrast, short-lived organisms have high influxes of susceptible individuals which lead to a large number of individuals in the population being susceptible to the pathogen. In addition to *Desmodus rotundus* having a longer lifespan, rabies virus infection in bats can be fatal, and this may be another reason for the increased level of pathogen reduction seen here in contrast to the rodent population with Lassa virus. Specifically, we found that increasing levels of virulence can increase the level of pathogen reduction that can be achieved, and suspect this to be because pathogen mortality leads to a decrease in the number of individuals in the population that vaccines may be wasted on. In addition, individuals shedding vaccine are more likely to survive and thus increase in frequency. See Fig D in S1 Text for more details.

## Discussion

We have used mathematical models of self-disseminating vaccines to evaluate how the timing and duration of vaccine distribution influences the impact of vaccination campaigns targeting seasonally fluctuating wildlife populations. Our results demonstrate that self-disseminating vaccines increase protection relative to traditional vaccines but that the magnitude of this increase can be sensitive to the timing of vaccine distribution. This is particularly true for transmissible vaccines that transmit only weakly (*R*_0,*V*_ < *R*_0,*P*_) and for transferable vaccines. Sensitivity to timing is most important for reservoir species with short lifespans and distinct birthing seasons. In these scenarios, it is generally best to distribute vaccine shortly after the peak of the reservoir birthing season. This general result mirrors previous findings for traditional, non self-disseminating wildlife vaccines from [23], but clarifies how the magnitude of the effect depends on the type of self-disseminating vaccine and its specific properties.

An important result that emerges from our work is that transferable vaccines are more sensitive to timing than are transmissible vaccines. This occurs primarily because transmissible vaccines can generate self-sustaining chains of transmission whereas transferable vaccines cannot. Thus, transferable vaccines can spread only to susceptible individuals at the time of vaccine introduction. In contrast, transmissible vaccines can be introduced earlier and yet still reach individuals that will be born later through persistent chains of vaccine transmission. This insensitivity to timing is greatest for highly contagious transmissible vaccines that generate long chains of transmission.

The importance of our results for real world applications depends on reservoir lifespan and the extent to which reservoir reproduction is seasonal. As demonstrated by our general and case study results, the lifespan of hosts has a large effect on the sensitivity to seasonality because it influences population turnover. For example, our results show that the success of attempts to vaccinate *M. natalensis*, the reservoir of Lassa virus, may be very sensitive to timing because the reservoir has a short lifespan. This sensitivity arises because rapid turnover within the reservoir population leads to a large, seasonal influx of susceptible individuals. In contrast, our results show that efforts to vaccinate the vampire bat, *D. rotundus*, are not particularly sensitive to timing due to the long lifespan of the reservoir. In long-lived populations like these, population turnover is low and the seasonal influx of newly born susceptible individuals is relatively small. Although we have illustrated the relevance of our general results using the specific examples of Lassa virus and rabies virus, these general results have broad implications for efforts to vaccinate reservoir animals against other important human pathogens. For instance, hantaviruses, such as Sin Nombre virus, also have reservoir species that are short-lived and exhibit seasonal reproduction [43]. In these cases, our results suggest that vaccination efforts will need to be well-timed and carefully planned to achieve maximum effectiveness.

Self-disseminating vaccines are promising tools for protecting human communities from spillover of emerging infectious diseases. The extent to which this promise is realized, however, may depend on biological nuances of particular reservoir species. For instance, our results assume reservoir individuals live in large well-mixed groups where individuals encounter one another at random. In contrast, many species live in smaller family groups or colonies where encounters between individuals outside of these groups are rare [44–47]. In these latter cases, vaccine spread beyond the group or colony of introduction may be significantly slowed. Our results also ignore age-structure which may lead to differences in the number of actively foraging, dispersing or allogrooming individuals in the population. These age-dependent behaviors could lead to different rates of vaccine uptake in young versus adult individuals as observed for uptake in oral vaccination campaigns of raccoon rabies [48]. Finally, our models have ignored the potential for maternal antibody transfer. Maternal antibodies can interfere with the vaccine and prevent juveniles from developing immunity to the pathogen [49]. Maternal antibodies in foxes, rodents, and bats have been seen to last 4–10 weeks [15, 50–52]. To avoid interference with maternal antibodies, vaccination may need to be delayed until antibodies wane. We suspect this may narrow the window of opportunity for effective vaccine distribution and make timing even more important than our results suggest.

In addition to assumptions about the biology of reservoir species, our models assume the vaccine is perfectly effective at blocking infection by the pathogen. This however, may not be the case and instead only a fraction of the time do individuals end up developing full immunity. Recent work from [37] suggests a transmissible vaccine against vampire bats with 70% efficacy would achieve a 20% lower level of outbreak reduction relative to a 100% efficacious vaccine. We suspect our models would produce comparable results for reductions in pathogen prevalence and that the importance of vaccine timing may be increased due to fewer successful vaccinations. We found similar results when we explored the effects of the number of vaccines distributed (Fig A in S1 Text).

Finally, our case study simulations have simplified the biology of the transmissible vaccine itself. Specifically, the betaherpesviruses currently proposed as vectors for the development of transmissible vaccines targeting Lassa virus and rabies virus are thought to alternate between periods of active shedding and latency [20, 37, 42]. In contrast, our models assume animals infected by the transmissible vaccine maintain active infections for the life of the animal. Although neglecting the detailed dynamics of vaccine latency and reactivation may alter the nuanced dynamics of vaccine spread, parameterizing our model using vaccine *R*_0_ guards against overestimating the effectiveness of the vaccine because this quantity captures transmission over the entire life of the infection.

However, these studies suggest *R*_0_ values of the transmissible vaccines to be significantly higher than what we explore in our results, therefore, we believe our results to be conservative estimates. If the transmissible vaccines in these hosts do have higher *R*_0_ values, we suspect our predictions about optimal timing would remain unchanged. In both case studies, a higher *R*_0_ value would make optimal timing insignificant despite host demography because the *R*_0_ of the vaccine would be substantially higher than the *R*_0_ of the pathogen.

Self-disseminating vaccines are in the early stages of development but their potential is extraordinary. Self-disseminating vaccines make wildlife vaccination campaigns more feasible by drastically reducing the level of vaccination coverage and effort needed to achieve pathogen elimination. However, self-disseminating vaccines have several scientific, regulatory, and societal hurdles to overcome before their implementation. When the time comes their implementation will need to be well thought out and guided to ensure their success and minimize risk. Modeling work allows us to investigate what is critical to the success of these vaccination campaigns while proof of concept and field studies are being developed. Previous work has already increased our understanding of these vaccines and has helped to guide development [16–20, 33, 37, 42, 53]. Our results add to this large body of work and further our understanding of the role that seasonality, lifespan, and vaccine characteristics play in the effectiveness of self-disseminating vaccination campaigns. Our results show that optimizing the timing and duration of vaccine delivery can make or break the success of a vaccination program in fluctuating wildlife populations. These results further demonstrate the importance of understanding the population ecology of wildlife species prior to implementing vaccination campaigns using self-disseminating vaccines.

## Supporting information

S1 Appendix Appendix. This document includes the full set of ordinary differential equations that make up the general models and case study models, as well as mathematical derivations of important values.
(PDF)

S1 Text Supporting information. This document includes additional figures for other parameters that were explored. Specifically, the number of vaccines distributed, rate at which the vaccine-laced gel is groomed off, pathogen recovery rates, virulence levels, interaction between lifespan and seasonality, and frequency-dependent transmission.
(PDF)

S1 Table Table of all parameter values used. To ensure the robustness of our general results we explored a large parameter space. This table describes the parameters used for various simulations. Specifically, we investigated the *R*_0_ of the vaccine and pathogen, the number of vaccines distributed, the lifespan of the reservoir host, the degree of seasonality of the host, length of vaccine infection, length of pathogen infection, pathogen-induced mortality, grooming rate of the gel (transferable vaccine only), and the interaction of lifespan and seasonality. In addition, this table includes the specific values used for the case studies. For each of these simulations we evaluated the average level of pathogen reduction across all possible times of vaccination and lengths of the vaccination campaign (see Methods in main text). Results of these simulations that were not in the main text can be found in S1 Text.
(TIFF)
