# Supplementary material for: Optimizing the delivery of self-disseminating vaccines in fluctuating wildlife populations
Source: PLoS Negl Trop Dis. 2023 Aug 18;17(8):e0011018. doi: 10.1371/journal.pntd.0011018 (PMC10468088; doi:10.1371/journal.pntd.0011018)
Supplement: S1 Appendix — This document includes the full set of ordinary differential equations that make up the general models and case study models, as well as mathematical derivations of important values. (PDF) [file pntd.0011018.s001.pdf]

Appendix for "Optimizing the delivery of self-disseminating vaccines in fluctuating wildlife populations"

Courtney L. Schreiner[1], Andrew J. Basinski[2], Christopher H. Remien[3], and Scott L. Nuismer[4]

[1]Department of Ecology and Evolutionary Biology, University of Tennessee Knoxville
[2]Institute for Interdisciplinary Data Sciences, University of Idaho
[3]Department of Mathematics and Statistical Science, University of Idaho
[4]Department of Biological Sciences, University of Idaho

July 26, 2023

1 Model Equations

1.1 General models

1.1.1 General transmissible vaccine model

$$\frac{dS}{dt} = b(t) - \beta_P SP - \beta_V SV - \sigma(t)\frac{S}{N} - dS \tag{1a}$$

$$\frac{dP}{dt} = \beta_P SP - \gamma_P P - vP - dP \tag{1b}$$

$$\frac{dV}{dt} = \beta_V SV + \sigma(t)\frac{S}{N} - \gamma_V V - dV \tag{1c}$$

$$\frac{dR}{dt} = \gamma_P P + \gamma_V V - dR. \tag{1d}$$

1.1.2 General transferable vaccine model

$$\frac{dS}{dt} = b(t) - \beta_P S(P+P_g) - \beta_g S(P_g+R_g) - \sigma(t)\frac{S}{S+P+R} - dS \tag{2a}$$

$$\frac{dP}{dt} = \beta_P S(P+P_g) - \sigma(t)\frac{P}{S+P+R} + \alpha N P_g - \gamma_P P + \gamma_g P_g - vP - dP \tag{2b}$$

$$\frac{dP_g}{dt} = \sigma(t)\frac{P}{S+P+R} - \alpha N P_g - \gamma_P P_g - \gamma_g P_g - vP_g - dP_g \tag{2c}$$

$$\frac{dR}{dt} = \beta_g S(P_g+R_g) - \sigma(t)\frac{R}{S+P+R} + \alpha N R_g + \gamma_P P + \gamma_g R_g - dR \tag{2d}$$

$$\frac{dR_g}{dt} = \sigma(t)\frac{S+R}{S+P+R} - \alpha N R_g + \gamma_P P_g - \gamma_g R_g - dR_g \tag{2e}$$

1.2 Case study models

1.2.1 Rodent case study model

For the Rodent case study we used the same models as the general models. See Eq 1 and Eq 2.

1.2.2 Bat case study model

For the Bat case study we used the same general framework except that instead of modeling density-dependent transmission of the vaccine and the pathogen we modeled frequency-dependent transmission. Discussion as to why this is can be found in the methods section. We therefore adjusted the models from Eq 1 and Eq 2 to be the following:

Transmissible vaccine model

$$\frac{dS}{dt} = b(t) - \frac{\beta_P SP}{N} - \frac{\beta_V SV}{N} - \sigma(t)\frac{S}{N} - dS \tag{3a}$$
$$\frac{dP}{dt} = \frac{\beta_P SP}{N} - \gamma_P P - vP - dP \tag{3b}$$
$$\frac{dV}{dt} = \frac{\beta_V SV}{N} + \sigma(t)\frac{S}{N} - \gamma_V V - dV \tag{3c}$$
$$\frac{dR}{dt} = \gamma_P P + \gamma_V V - dR. \tag{3d}$$

Transferable vaccine model

$$\frac{dS}{dt} = b(t) - \frac{\beta_P S(P+P_g)}{N} - \frac{\beta_g S(P_g+R_g)}{N} - \sigma(t)\frac{S}{S+P+R} - dS \tag{4a}$$
$$\frac{dP}{dt} = \frac{\beta_P S(P+P_g)}{N} - \sigma(t)\frac{P}{S+P+R} + \alpha P_g - \gamma_P P + \gamma_g P_g - vP - dP \tag{4b}$$
$$\frac{dP_g}{dt} = \sigma(t)\frac{P}{S+P+R} - \alpha P_g - \gamma_P P_g - \gamma_g P_g - vP_g - dP_g \tag{4c}$$
$$\frac{dR}{dt} = \frac{\beta_g S(P_g+R_g)}{N} - \sigma(t)\frac{R}{S+P+R} + \alpha R_g + \gamma_P P + \gamma_g R_g - dR \tag{4d}$$
$$\frac{dR_g}{dt} = \sigma(t)\frac{S+R}{S+P+R} - \alpha R_g + \gamma_P P_g - \gamma_g R_g - dR_g \tag{4e}$$

2 Setting the birth scaling constant k

In our simulations, the scaling constant k in the birthing function is determined by the user-specified values d, s, and $\bar{N}$. To solve for the value of k, we first rewrite the birthing function as

$$b(t) = k \cdot e^{-s\cdot\sin^2\left(\frac{\pi}{365}\cdot(t)\right)} \tag{5}$$
$$= k\bar{b}(t). \tag{6}$$

The differential equation that describes the host population size in the absence of any infectious agent is

$$\frac{dN}{dt} = b(t) - dN. \tag{7}$$

Let $N^*(t)$ denote the T-periodic solution of Eq (7) with mean value $\bar{N}$. Then

$$\frac{1}{T}\int_0^T N^*(t)\,dt = \bar{N}. \tag{8}$$

This implies

$$\frac{dN^*}{dt} = b(t) - dN^* \tag{9}$$

$$\int_0^T \frac{dN^*}{dt}\,dt = \int_0^T b(t)\,dt - d\int_0^T N^*\,dt \tag{10}$$

$$0 = k\int_0^T \bar{b}(t)\,dt - dT\bar{N}. \tag{11}$$

The left hand side of Eq (11) is zero because N^* is T-periodic. Thus, we have

$$k = \frac{dT\bar{N}}{\int_0^T \bar{b}(t)\,dt} \tag{12}$$

Thus, for a specified d, s, T, and $\bar{N}$, Eq (12) can be numerically integrated to solve for the implied value of k.

3 Derivation of R_0

In this section, we derive an expression for the basic reproduction number, notated R_0, that describes the average number of new infections that result when a single infected individual is introduced at a random time into a stably cycling population of susceptible hosts. We keep our derivation broad so as to simultaneously derive the relevant R_0 for the pathogen, transmissible vaccine, and transferable vaccine, under both density and frequency-dependent transmission.

Let $N^*(t)$ denote the T-periodic limit cycle that describes a population of susceptible hosts in the absence of infection and vaccination. We assume that $N^*(t) >> 1$ so that the susceptible population is not significantly depleted by the infection process. Let β, γ, v denote the transmission rate, recovery rate, and the virulence rate of the infectious agent. Let $C(N)$ describe how the per-capita rates of host interaction scale with population size: under a density-dependent scenario, $C(N) = N$, while under a frequency-dependent scenario, $C(N) = 1$ [1]. For the transferable vaccine, we assume that grooming interactions scale with population size in the same way as infectious contacts. Thus, $\alpha C(N)$ describes the rate at which vaccine is groomed off gelled individuals in a population of size N.

When a single infected host is introduced into a susceptible population described by $N^*(t)$, the rate of new infections at time t is $\beta C(N^*)$. Here, we omit the dependence of N^* on t to simplify notation. Depending on the infectious agent being described, this infection rate continues until the initial infected host dies due to natural mortality (at rate d), dies due to pathogen virulence (at rate v), recovers from infection (at rate γ), or in the transferable vaccine case, leaves the infectious class due to grooming of gel at rate $\alpha C(N^*)$. Note that $\alpha = 0$ in the case of the pathogen or transmissible vaccine.

Let t_0 denote the time at which the infected individual is introduced. The total number of new infections caused by the infected individual is obtained by integrating the infection rate ($\beta C(N^*)$) multiplied by the probability that the individual has not recovered or died from time $t = t_0$ to time $t = \infty$.

To find the probability that the individual has not lost infectiousness status, let $P(t,t_0)$ denote the probability that the individual is still infectious at time $t > t_0$. We assume that $P(t,t_0)$ is described by a Poisson process with probabilistic rates at which infectiousness is lost due to natural death (d), degradation of vaccine or recovery (γ), mortality due to pathogen virulence (ν), or grooming ($\alpha C(N^*)$). Then for time $t > t_0$, and a small time interval Δt, $P(t,t_0)$ satisfies

$$P(t+\Delta t,t_0) = P(t,t_0)\,(1-\Delta t(d+\gamma+\nu+\alpha C(N^*))) + O(\Delta t^2) \tag{13}$$

with initial condition $P(t_0,t_0) = 1$. Here, $O(\Delta t^2)$ denotes terms in Eq (13) that become negligible in the limit as Δt approaches zero. In words, Eq (13) describes how the probability of the individual still being infectious at time $t+\Delta t$ is approximately equal to the probability that the individual was infectious at time t, multiplied by the probability that the individual's infectious status has not changed in the interval $(t,t+\Delta t)$.

By rearranging terms in Eq (13) and taking the limit as Δt approaches zero, we derive the continuous time differential equation

$$\frac{\partial P}{\partial t} = -P(t,t_0)\ (d+\gamma+\nu+\alpha C(N^*)). \tag{14}$$

Dividing both sides of Eq (14) by $P(t,t_0)$ and integrating over t from time t_0 yields the probability that an initial infected individual introduced at time t_0 is still capable of infecting others at time t:

$$P(t,t_0) = \mathrm{Exp}\left[-(d+\gamma+\nu)(t-t_0) - \alpha\int_{t_0}^{t} C(N^*(s))\,ds\right], \tag{15}$$

where Exp[x]= e^x denotes the exponential function.

With Eq (15) in hand, we can express the total number of new infections caused by the introduced infected individual as

$$\int_{t_0}^{\infty} \beta C(N^*(t))\,\mathrm{Exp}\left[-(d+\gamma+\nu)(t-t_0) - \alpha\int_{t_0}^{t} C(N^*(s))\,ds\right] \mathrm{d}t. \tag{16}$$

Eq (16) highlights that, because the population size $N^*(t)$ is non-constant, the number of new infections is a function of the time t_0 at which the infected individual is introduced. In order to find the average number of new infections generated by an infected that is introduced at a randomly chosen time, we integrate Eq (16) with respect to t_0 over the interval $[0,T]$, and divide by $\frac{1}{T}$. Note that because $N^*(t)$ is T-periodic, averaging over introduction times that are outside the interval $[0,T]$ is redundant. Consequently, we have

$$R_0 = \frac{1}{T}\int_0^T \int_{t_0}^{\infty} \beta C(N^*(t))\,\mathrm{Exp}\left[-(d+\gamma+\nu)(t-t_0) - \alpha\int_{t_0}^{t} C(N^*(s))\,ds\right] \mathrm{d}t\,\mathrm{d}t_0. \tag{17}$$

3.1 Transferable vaccine and density-dependent scaling of host interactions

In the case of the transferable vaccine and density dependent host interactions, virulence is absent so we set $v = 0$. In addition, $\alpha \neq 0$ and $C(N^*) = N^*$ so the integral described by Eq (17) is difficult to simplify by the presence of the antiderivative of $N^*(t)$ in the exponent.

3.2 Transmissible vaccine and pathogen or frequency-dependent scaling of host interactions

In the case of the transmissible vaccine and the pathogen, or when interactions are frequency dependent, the expression for R_0 in Eq (17) can be simplified. In all of these cases, the double integral described by Eq (17) can be simplified by using the change of coordinates $u = t$, $w = t - t_0$. This change of coordinates needs to be applied to three terms in the above integral: the area differential $\mathrm{d}t\,\mathrm{d}t_0$, the limits of integration, and the integrand [2].

Let $X(u,w) = (u, u-w)$ denote the vector-valued function that converts (u,w) coordinates into (t,t_0) coordinates. Then the area differential $\mathrm{d}t\,\mathrm{d}t_0$ is equal to $|DX|\,\mathrm{d}u\,\mathrm{d}w$, where D denotes the Jacobian operator with respect to u and w, and $|\cdot|$ denotes the determinant. Because $|DX| = 1$, we have $\mathrm{d}t\,\mathrm{d}t_0 = \mathrm{d}u\,\mathrm{d}w$. The region of integration in the (u,w) plane can be found by drawing the region of integration in the (t,t_0) plane, and identifying boundary lines with their analogue in the (u,w) plane (Fig 1). Finally, the integrand is transformed by the substitution $t \to u$ and $t - t_0 \to w$.

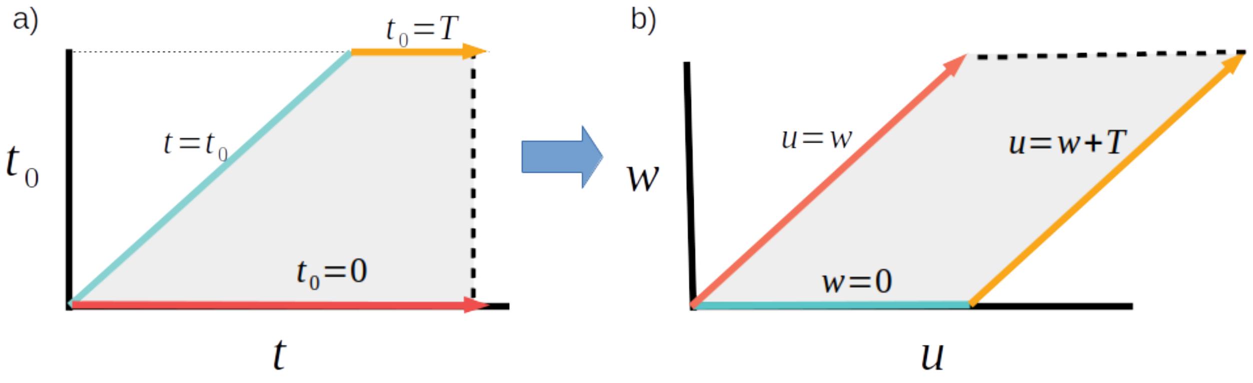

Figure 1: Region of integration (gray) of Eq (17) in the (t,t_0) plane (a). When $\alpha = 0$, the calculation of R_0 is simplified by transforming the region into the (u,w) plane (b). The dashed boundary lines indicate that the region continues out to infinity. Boundary lines and their transforms are identified by the same color.

We first evaluate the case when host interactions are density-dependent ($C(N) = N$). When $\alpha = 0$ these substitutions allow us to transform the integral in Eq. (17) and evaluate as follows:

$$R_0 = \frac{1}{T}\int_0^\infty \int_w^{w+T} \beta N^*(u)\, e^{-(d+\gamma+v)w}\mathrm{d}u\,\mathrm{d}w \tag{18}$$

$$= \frac{\beta}{T}\int_0^\infty \left(\int_w^{w+T} N^*(u)\,\mathrm{d}u\right) e^{-(d+\gamma+v)w}\mathrm{d}w \tag{19}$$

$$= \beta \bar{N}^* \int_0^\infty e^{-(d+\gamma+v)w}\mathrm{d}w \tag{20}$$

$$= \frac{\beta \bar{N}^*}{d+\gamma+v}. \tag{21}$$

Here, $\bar{N}^*$ denotes the average population size over a single period T. Virulence v is possibly nonzero for the pathogen's R_0, and virulence is set to zero for the transmissible or transferable vaccines' R_0's.

When the rate of interactions is frequency-dependent and for general $\alpha \geq 0$, the sequence of equations 18 - 21 can be applied in a similar manner to obtain

$$R_0 = \frac{\beta}{d+\gamma+v+\alpha}. \tag{22}$$

3.3 Setting the transmission rate β

Equations (17), (21), and (22) are used to define the transmission rate β that corresponds to specific values of R_0 in our simulations. For a given simulation and infectious agent, we define an average population size $\bar{N}$, death rate d, virulence v, recovery rate γ, gel grooming rate α, and basic reproduction number R_0. In the case of a density dependence and for the transmissible vaccine or pathogen, Eq (21) can then be used to solve for the value of β that is implied by the user-defined parameters. If the host interaction rate is frequency dependent, Eq (22) is used to derive β.

The density dependent, transferable vaccine case is more difficult because we need the solution of $N^*(t)$ to evaluate Eq (17). To this end, we first solve for the value of k using Eq (12) and parameters specified by the user. k, in turn, is used to define the birthing rate $b(t)$. Next, we obtain a numerical approximation of $N^*(t)$ by simulating the population equation Eq (7). Specifically, we simulate Eq (7) for 10 years to allow the solution to converge to the stable limit cycle $N^*(t)$. Next, we use the function "approxfun" in R to approximate the stable limit cycle $N^*(t)$. Finally, we use these numerical approximations to evaluate the double integral of Eq (17) and solve for the value of β that is implied by a user specified R_0. All integration was performed in the statistical language R using the deSolve package [3].

4 Estimating seasonality parameter for case studies

In this section, we describe how the seasonality parameter s was parameterized for the case studies on *Mastomys natalensis* and *Desmodus rotundus*.

4.1 *Mastomys natalensis*

We use data on trapping success of *M. natalensis* in Guinea to broadly estimate the seasonality parameter s [4]. This study contains time series of trap success from two towns. Because *M. natalensis* is typically associated with human habitation, we use the within-house trap success as a relative measure of *M. natalensis* population size. We choose a value of s so that, when the average population size in our simulation is 2000 rodents, the ratio of the maximum and minimum population size from our model matches the ratio of the maximum to minimum trap success from these time series data. Fig 2 of the study implies that this ratio is approximately two [4]. With this ratio in hand, we use the "optimize" function in R and the population demography model

described by Eq (7) to find the value of s that minimizes the squared error between the simulated maximum:minimum population ratio (after a 101 year burn-in period) and the estimated true ratio of two. This method yields a value of $s = 13.078$.

4.2 *Desmodus rotundus*

To parameterize the birth function for *Desmodus rotundus*, we choose a value of the seasonality parameter s that matches the ratio of the maximum birth rate to the minimum birth rate. We do this by using the analytical form of the maximum and minimum of the birth function. For a given year n (a positive integer), the function $b(t)$ reaches its seasonal maximum k at $t = 365n$ and minimum $k \cdot \exp{-s}$ when $t = \frac{365}{2} + 365n$. We then use the ratio of the maximum and minimum birth rate, respectively referred to as "max" and "min", to estimate the seasonality parameter s:

$$\frac{\text{max}}{\text{min}} = \frac{k}{k \cdot e^{-s}} \tag{23}$$

$$\frac{\text{max}}{\text{min}} = \frac{1}{e^{-s}} \tag{24}$$

$$\frac{\text{max}}{\text{min}} = e^{s} \tag{25}$$

Thus, we find

$$s = \log\left(\frac{\text{max}}{\text{min}}\right) \tag{26}$$

With Eq (26) in hand, we can use data on the estimates of birth rate throughout the year to estimate s.

Estimates for birth rates for *Desmodus rotundus* have come from data on lactating females. Specifically, studies in Argentina on vampire bats found a direct relationship between the number of lactating females and the number of births in the population [5]. We use estimates and the method outlined above to find an estimate for s. Based on Fig 1 from [5] and Fig S1 from [6] we estimate that the ratio of the maximum:minimum birth rate is 40:3. With these values, Eq (26) implies $s = 2.59$.

References

[1] Keeling MJ, Rohani P. Modeling infectious diseases in humans and animals. Princeton University Press; 2011.

[2] Stewart J. Essential calculus: Early transcendentals. Cengage Learning; 2012.

[3] Soetaert K, Petzoldt T, Setzer RW. Solving Differential Equations in R: Package deSolve. J Stat Softw. 2010;33(9):1–25. doi:https://doi.org/10.18637/jss.v033.i09.

[4] Fichet-Calvet E, Lecompte E, Koivogui L, Soropogui B, Doré A, Kourouma F, et al. Fluctuation of abundance and Lassa virus prevalence in *Mastomys natalensis* in Guinea, West Africa. Vector-Borne and Zoonotic Diseases. 2007;7(2):119–128.

[5] Lord RD, Muradali F, Lazaro L. Age composition of vampire bats (*Desmodus rotundus*) in northern Argentina and southern Brazil. Journal of Mammalogy. 1976;57(3):573–575.

[6] Blackwood JC, Streicker DG, Altizer S, Rohani P. Resolving the roles of immunity, pathogenesis, and immigration for rabies persistence in vampire bats. Proceedings of the National Academy of Sciences. 2013;110(51):20837–20842.
