# Supplementary material for: Optimizing the delivery of self-disseminating vaccines in fluctuating wildlife populations
Source: PLoS Negl Trop Dis. 2023 Aug 18;17(8):e0011018. doi: 10.1371/journal.pntd.0011018 (PMC10468088; doi:10.1371/journal.pntd.0011018)
Supplement: S1 Text — This document includes additional figures for other parameters that were explored. Specifically, the number of vaccines distributed, rate at which the vaccine-laced gel is groomed off, pathogen recovery rates, virulence levels, interaction between lifespan and seasonality, and frequency-dependent transmission. (PDF) [file pntd.0011018.s002.pdf]

Supplemental

Courtney L Schreiner, Andrew J Basinski, Christopher H Remien, Scott L Nuismer

07-12-2023

Supplemental

Provided here is the supplemental material for the manuscript by Schreiner et. al. titled "Optimizing the delivery of self-disseminating vaccines in fluctuating wildlife populations".

General simulations

Number of Vaccines

Here we explored how the number of vaccines affected the importance of timing vaccination as well as the overall level of pathogen reduction achieved. In the figure below the R_0 of the vaccine ($R_{0,V}$) is 1.5 while the R_0 of the pathogen ($R_{0,P}$) is 2. Additionally, the average population size is 2000. Important conclusions we can draw from this figure are that increasing the number of vaccines increases the overall level of pathogen reduction that can be achieved for both the transmissible and transferable vaccine. Timing vaccine delivery is more important with fewer vaccines because it becomes increasingly important not to waste vaccines by vaccinating during times of year where there are few individuals.

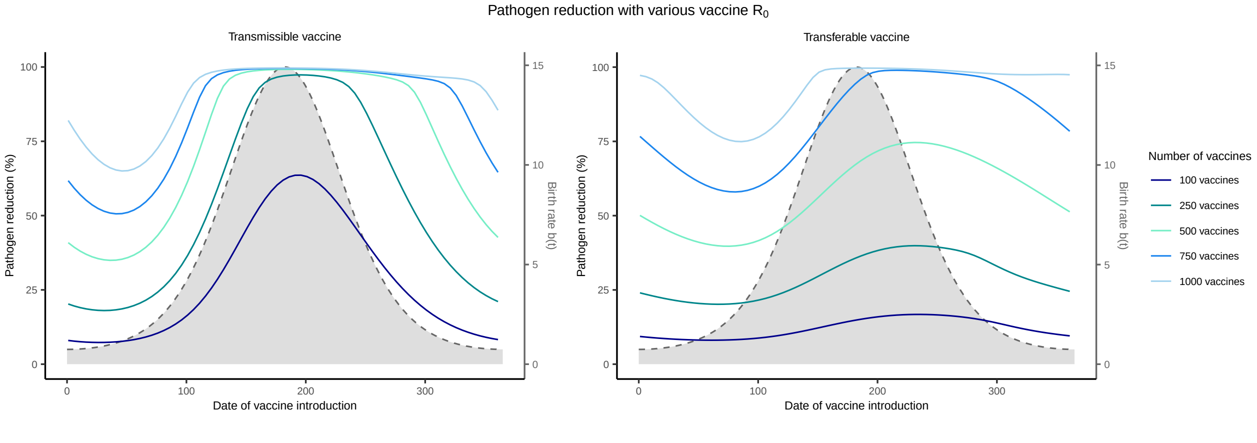

Figure A: Level of pathogen reduction achieved across various times of vaccination with different levels of vaccination coverage indicated by the different colors. Solid lines represent the level of pathogen reduction achieved for a given date of vaccine introduction. The grey region outlined by the dashed lined represents the seasonal birthing season where day 1 corresponds to the first day of the birthing season. Parameters used were, $\overline{N} = 2000$, $s = 3$, $d = 1/365$, $R_{0,P} = 2$, $R_{0,V} = 1.5$, $N_v = (100, 250, 500, 750, 1000)$, $\gamma_V = 1/21$,$\gamma_g = 1/21$, $\gamma_P = 1/21$, $\alpha = 1/15000$, $\nu = 0$.

Different alpha values

A parameter that we explored that is exclusive to the transferable vaccine is the rate at which gel is groomed off of individuals (α). As we increase α we see increased levels of pathogen reduction. This is because when the gel stays on longer there is a greater opportunity to achieve additional transfers of gel that lead to immunity. However, this increase is minimal because individuals that have gel on them may leave the gelled class due to the gel losing its infectiousness (γ_g.

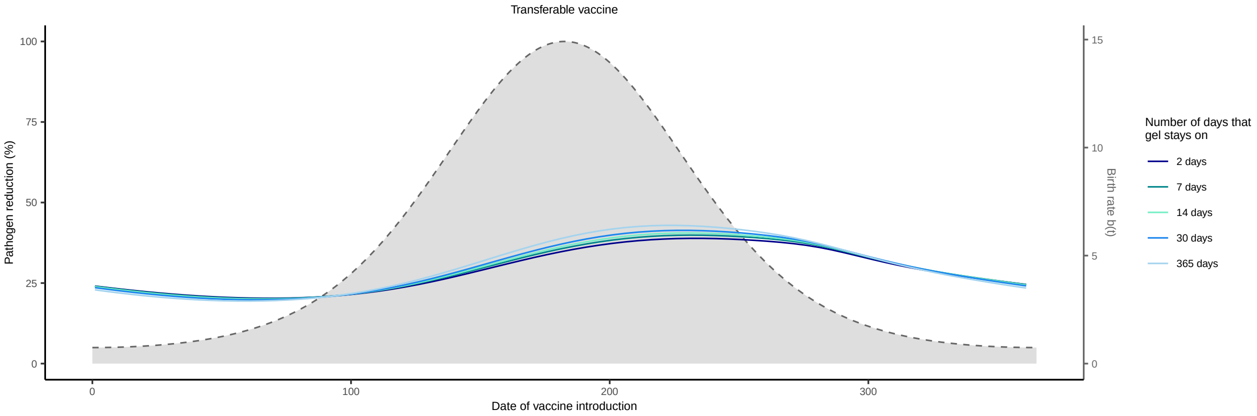

Figure B: Level of pathogen reduction achieved across various times of vaccination with different grooming rates indicated by the different colors. The grooming rate controls the average length of time it takes for gel to be removed via grooming. Solid lines represent the level of pathogen reduction achieved for a given date of vaccine introduction. The grey region outlined by the dashed lined represents the seasonal birthing season where day 1 corresponds to the first day of the birthing season. Parameters used were, $\overline{N} = 2000$, $s = 3$, $d = 1/365$, $R_{0,P} = 2$, $R_{0,V} = 1.5$, $N_v = 250$, $\gamma_g = 21^{-1}$, $\gamma_P = 21^{-1}$, $\alpha = (2^{-1}, 7^{-1}, 14^{-1}, 30^{-1}, 365^{-1})$, $\nu = 0$.

Different pathogen recovery rates

Pathogens vary in the duration of infection and this could potentially affect the optimal time to distribute vaccines. Our results indicate that in general there is more opportunity for pathogen reduction with acute infections than with long-term infections. This is because with long-term infections in seasonally fluctuating populations, pathogen prevalence does not fluctuate to the same degree as with acute infections. With lower fluctuations in pathogen prevalence there is a smaller proportion of the population that are susceptible, thus the vaccine achieves lower levels of pathogen reduction. In contrast, with acute infections, there are times of year where pathogen prevalence is low and the vaccine can spread before the pathogen takes hold, and this leads to higher levels of pathogen reduction.

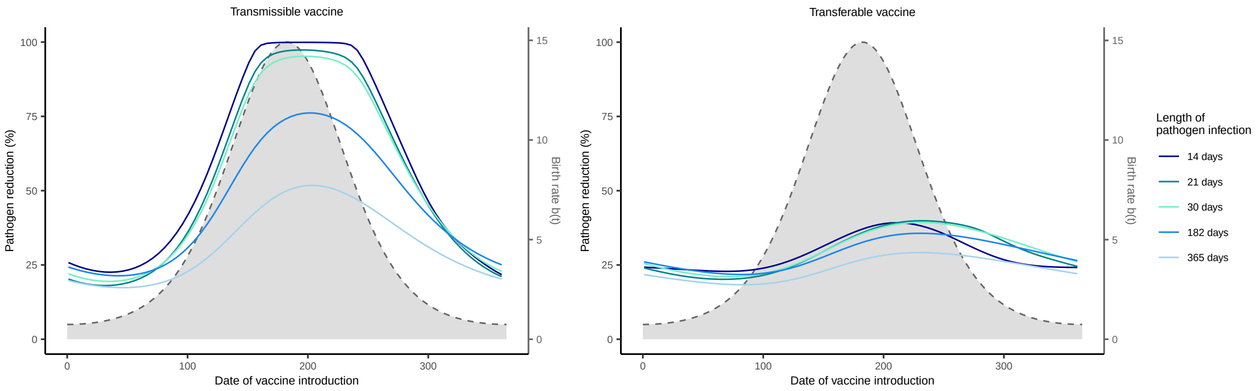

Figure C: Level of pathogen reduction achieved across various times of vaccination with different pathogen recovery rates indicated by the different colors. This compares vaccination strategies across different types of infections. For example, Acute vs chronic infections. Solid lines represent the level of pathogen reduction achieved for a given date of vaccine introduction. The grey region outlined by the dashed lined represents the seasonal birthing season where day 1 corresponds to the first day of the birthing season. Parameters used were, $\overline{N} = 2000$, $s = 3$, $d = 1/365$, $R_{0,P} = 2$, $R_{0,V} = 1.5$, $N_v = 250$, $\gamma_V = 1/21$, $\gamma_g = 1/21$, $\gamma_P = (14^{-1}, 21^{-1}, 30^{-1}, (365/2)^{-1}, 365^{-1})$, $\alpha = 1/15000$, $\nu = 0$.

Different virulence levels

Several infectious pathogens can be fatal to the hosts that carry them. Here we explore a range of virulence levels to investigate the affect pathogen induced mortality may have on optimal timing and pathogen reduction. We found that higher levels of pathogen reduction can be achieved with higher virulence levels for both the transmissible and transferable vaccine. Higher virulence leads to individuals leaving the system, specifically reducing the number of pathogen infected individuals, this in turn reduces pathogen prevalence and reduces the number of vaccines that are wasted on non-susceptible individuals in the population. In addition, the individuals that receive vaccine are more likely to survive and subsequently increase the proportion of vaccinated individuals. Optimal timing of vaccine distribution is not affected for either of the self-disseminating vaccines. There are some sharp drops of pathogen reduction in our output for the transferable vaccine for the higher levels of virulence. We suspect this is due to numerical error with such high virulence rates.

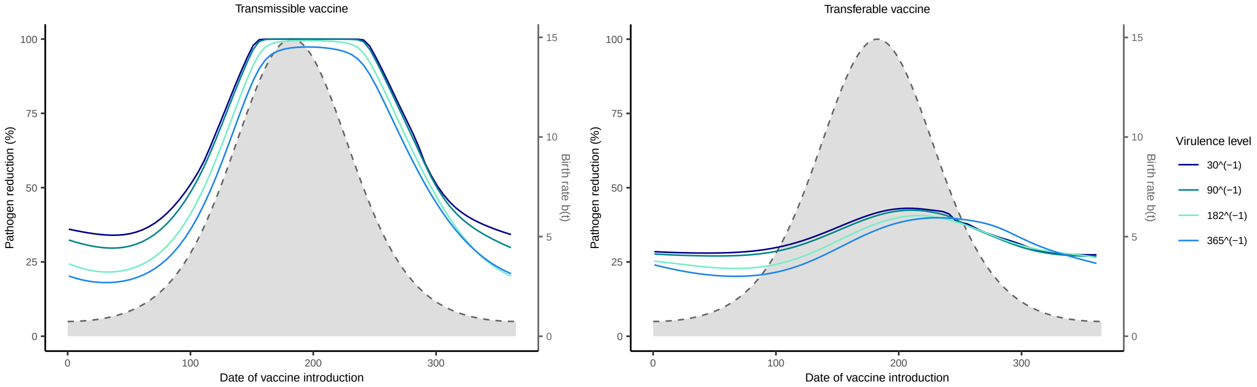

Figure D: Level of pathogen reduction achieved across various times of vaccination with different virulence levels indicated by the different colors. Solid lines represent the level of pathogen reduction achieved for a given date of vaccine introduction. The grey region outlined by the dashed lined represents the seasonal birthing season where day 1 corresponds to the first day of the birthing season. Parameters used were, $\overline{N} = 2000$, $s = 3$, $d = 1/365$, $R_{0,P} = 2$, $R_{0,V} = 1.5$, $N_v = 250$, $\gamma_V = 1/21$, $\gamma_g = 1/21$, $\gamma_P = 1/21$, $\alpha = 1/15000$, $\nu = (14^{-1}, 30^{-1}, 90^{-1}, (365/2)^{-1}, 365^{-1})$.

Lifespan versus seasonality

Host lifespan and seasonality affect the birthing season. We decided to explore several combinations of host lifespan and seasonality to see how timing of vaccination and the level of pathogen reduction possible were affected. As discussed in the main text, hosts with longer lifespans are less sensitive to timing while hosts with short lifespans are more sensitive to timing. In hosts with short lifespans, seasonality can increase the importance of timing and the level of pathogen reduction that can be achieved. What is illustrated here is that hosts with short lifespans have much higher overall birth rates. This leads to a large influx of susceptible individuals in the population which causes a reduction in the proportion of immune individuals. This creates a situation where the population is vulnerable to the pathogen and vaccines must be well timed in order to protect the population from being overtaken by the pathogen. In contrast, hosts with longer lifespans have lower overall birth rates so there is not a large influx in susceptible individuals, additionally, the individuals that you vaccinated in the prior year are still present due to long lifespan. This leads to the population maintaining a high proportion of immunity throughout the year. Thus, distributing vaccine during different times of the year relative to the birthing season does not make a significant difference. Even when seasonality is high, the peak birth rate during the birthing season is not large enough to lead to a significant increase of susceptible individuals so timing does not matter in hosts with long lifespans regardless of the degree of seasonality. Timing is most important for hosts with short lifespans, and even more critical for hosts with short lifespans and distinct birthing seasons.

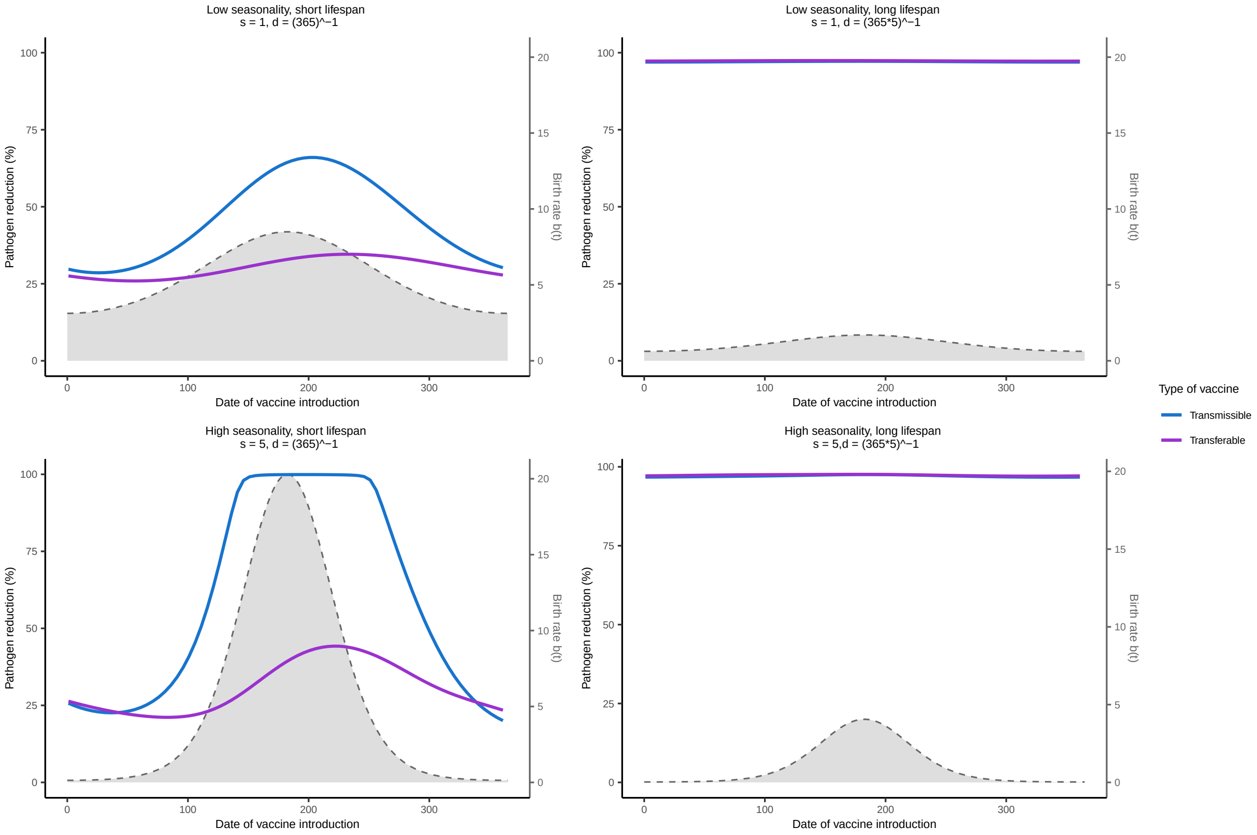

Figure E: Level of pathogen reduction achieved across various times of vaccination with different host demography combinations. We looked at when hosts had low seasonality and short lifespans (top left), low seasonality and long lifespans (top right), high seasonality and short lifespans (bottom left), or high seasonality and long lifespans (bottom right). Solid lines represent the level of pathogen reduction achieved for a given date of vaccine introduction for either the transmissible vaccine in blue or the transferable vaccine in purple. The grey region outlined by the dashedlines represent the seasonal birthing season. Day 1 corresponds to the first day of the birthing season as well as the first possible day of vaccine introduction. Parameters used were, $\overline{N} = 2000$,($s = 1$ & $d = 1/365$, $s = 1$ & $d = 1/365 \times 5$, $s = 5$ & $d = 1/365$, $s = 5$ & $d = 1/365 \times 5$), $R_{0,P} = 2$, $R_{0,V} = 1.5$, $N_v = 250$, $\gamma_V = 1/21$, $\gamma_g = 1/21$, $\gamma_P = 1/21$, $\alpha = 1/15000$, $\nu = 0$.

Frequency-dependent case:

Some pathogens spread via frequency-dependent transmission (FD) while others spread via density-dependent transmission (DD) although there may be other transmission routes that lie somewhere between these two forms. The different modes of transmission can have effects on pathogen dynamics thus we compared the level of pathogen reduction achieved across various times of vaccine introduction for density-dependent (dotted line) and frequency-dependent (solid lines) transmission. We found that higher levels of pathogen reduction can be achieved under density-dependent transmission compared to frequency-dependent transmission. If we look at when $R_{0,V} = 1.5$, we have a situation where the transmissible vaccine can capitalize on the large increase in the number of susceptible individuals in the population, and out-compete the pathogen if timed correctly under density-dependent transmission. However, under frequency-dependent transmission, there is only a 75% reduction in pathogen prevalence. This stark difference is due to these routes of transmission. Under density-dependent transmission the large increase in susceptible individuals helps the vaccine spread more effectively. Whereas under frequency-dependent transmission, the large increases in population size doesn't have as strong of an effect. For frequency-dependent transmission, what matters for optimal timing is when the proportion of susceptible individuals has peaked. This will lag

behind the number of individuals since the number of susceptible individuals can change rapidly whereas the proportion of susceptible individuals changes slowly. This is also the reason for less peaked optimal times of vaccine introduction under frequency-dependent transmission. Overall, frequency-dependent versus density-dependent transmission has little effect on our qualitative results.

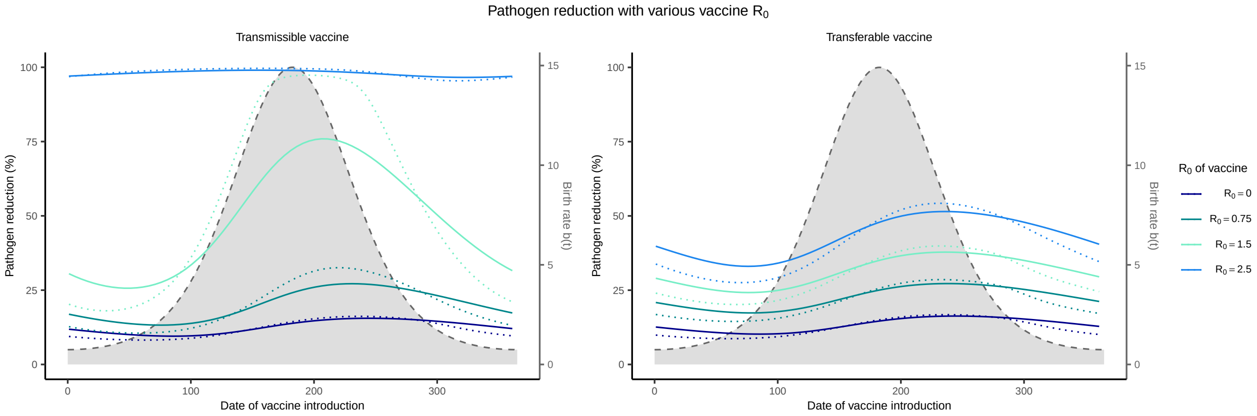

Figure F: Level of pathogen reduction achieved across various times of vaccination with different vaccine R_0 indicated by the different colors. Solid lines represent the level of pathogen reduction achieved for a given date of vaccine introduction under frequency-dependent transmission. Dotted lines are density-dependent transmission. The grey region outlined by the dashed lined represents the seasonal birthing season where day 1 corresponds to the first day of the birthing season. Parameters used were, $\overline{N} = 2000$, $s = 3$, $d = 1/365$, $R_{0,P} = 2$, $R_{0,V} = (0, 0.75, 1.5, 2)$, $N_v = 250$, $\gamma_V = 1/21$, $\gamma_{=}$1/21, $\gamma_P = 1/21$, $\alpha = 1/15000$, $\nu = 0$.
