# Supplementary figures and images for: Optimizing the delivery of self-disseminating vaccines in fluctuating wildlife populations
Source: PLoS Negl Trop Dis. 2023 Aug 18;17(8):e0011018. doi: 10.1371/journal.pntd.0011018 (PMC10468088; doi:10.1371/journal.pntd.0011018)

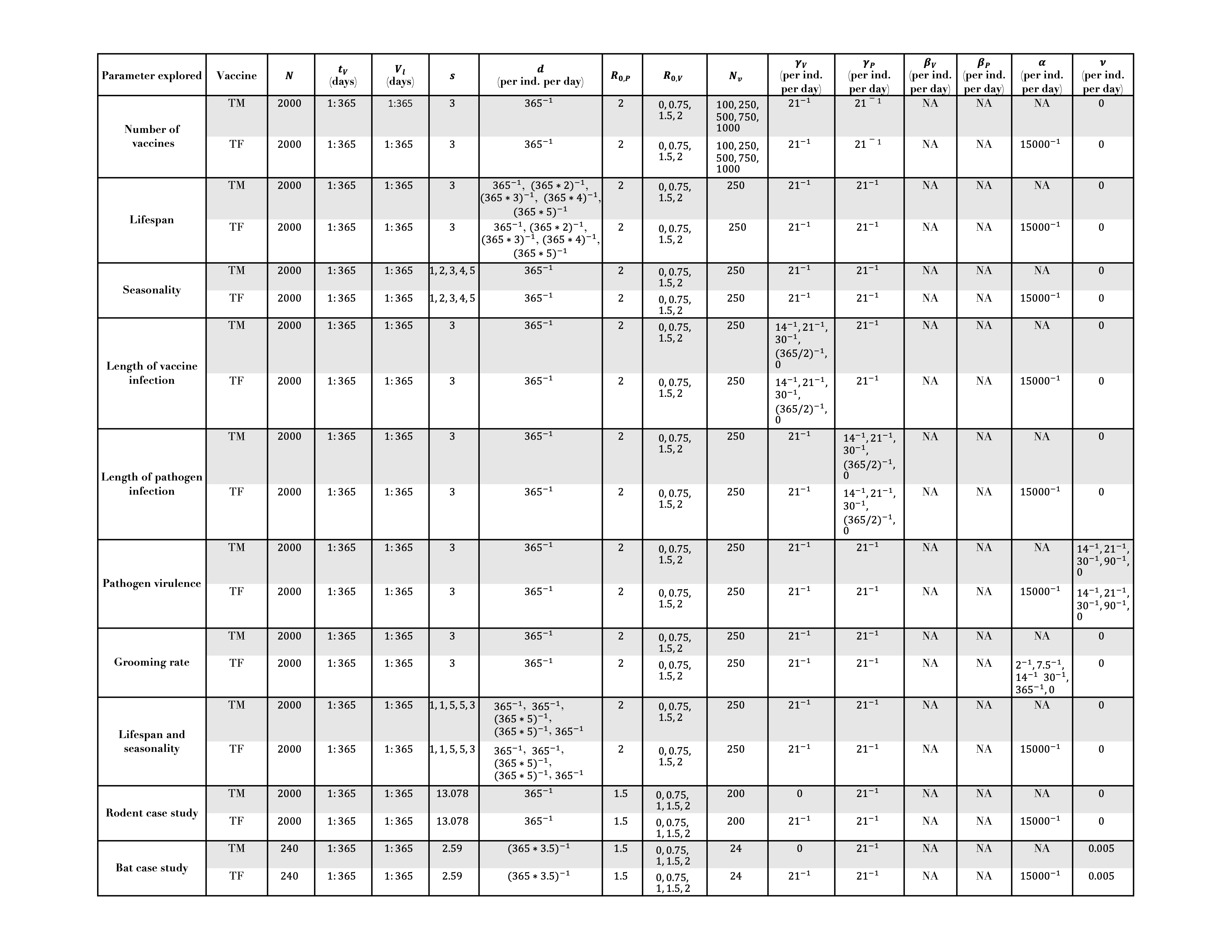

Supplement: S1 Table — To ensure the robustness of our general results we explored a large parameter space. This table describes the parameters used for various simulations. Specifically, we investigated the R0 of the vaccine and pathogen, the number of vaccines distributed, the lifespan of the reservoir host, the degree of seasonality of the host, length of vaccine infection, length of pathogen infection, pathogen-induced mortality, grooming rate of the gel (transferable vaccine only), and the interaction of lifespan and seasonality. In addition, this table includes the specific values used for the case studies. For each of these simulations we evaluated the average level of pathogen reduction across all possible times of vaccination and lengths of the vaccination campaign (see Methods in main text). Results of these simulations that were not in the main text can be found in S1 Text. (TIFF) [file pntd.0011018.s003.tiff]
